# Doxycycline promotes proteasome fitness in the central nervous system

**DOI:** 10.1038/s41598-021-96540-z

**Published:** 2021-08-20

**Authors:** Edmund Charles Jenkins, Matthew J. O’Connell, Giovanni Manfredi, Doris Germain

**Affiliations:** 1 Division of Hematology/Oncology, Department of Medicine, Icahn School of Medicine at Mount Sinai, Tisch Cancer Institute, New York, NY 10029 USA; 2 Department of Oncological Sciences, Icahn School of Medicine at Mount Sinai, Tisch Cancer Institute, New York, NY 10029 USA; 3 Feil Family Brain and Mind Research Institute, Weill Cornell Medicine, New York, NY 10065 USA

**Keywords:** Mitochondria, Proteolysis

## Abstract

Several studies reported that mitochondrial stress induces cytosolic proteostasis in yeast and *C. elegans*. Notably, inhibition of mitochondrial translation with doxcycyline decreases the toxicity of β-amyloid aggregates, in a *C. elegans*. However, how mitochondrial stress activates cytosolic proteostasis remains unclear. Further whether doxycycline has this effect in mammals and in disease relevant tissues also remains unclear. We show here that doxycycline treatment in mice drastically reduces the accumulation of proteins destined for degradation by the proteasome in a CNS region-specific manner. This effect is associated with the activation of the ERα axis of the mitochondrial unfolded protein response (UPR^mt^), in both males and females. However, sexually dimorphic mechanisms of proteasome activation were observed. Doxycycline also activates the proteasome in fission yeast, where ERα is not expressed. Rather, the ancient ERα-coactivator Mms19 regulates this response in yeast. Our results suggest that the UPR^mt^ initiates a conserved mitochondria-to-cytosol stress signal, resulting in proteasome activation, and that this signal has adapted during evolution, in a sex and tissue specific-manner. Therefore, while our results support the use of doxycycline in the prevention of proteopathic diseases, they also indicate that sex is an important variable to consider in the design of future clinical trials using doxycycline.

## Introduction

Several studies have reported that different mitochondrial stresses induce a cross talk between the mitochondria and cytosolic proteostasis in a variety of organisms, including an attenuation of protein translation^1,2^, increased expression of cytosolic heat shock proteins and chaperones^3,4^, and alteration in proteasome activity^5–7^. In yeast, reduction in mitochondrial proteins import, and therefore accumulation of mitochondrial protein precursors in the cytosol, induces the unfolded protein response activated by mistargeting of proteins (UPR^am^), which promotes the activity of the proteasome^7^. Further, a recent study reported that treatment of *C. elegans* with doxycycline, which inhibits mitochondrial translation^8–11^ reduces the accumulation of amyloid-β in the cytoplasm, a protein associated with Alzheimer’s disease^12^. Collectively, these studies suggest that mitochondrial stress-mediated regulation of cytosolic proteostasis and proteasome activity may be an evolutionary conserved adaptation aimed at maintaining cellular protein homeostasis.

The mitochondrial unfolded protein response (UPR^mt^) is a complex retrograde signaling cascade involving several distinct axes, which is now more broadly considered to be part of the integrated mitochondrial stress response^13^. While attenuation of protein translation was clearly shown to be dependent on the CHOP/ATF4/ATF5 axis of the UPR^mt^^1,13^ the activation of cytosolic heat shock proteins and the elimination of toxic proteins aggregates were found to be independent of this axis^3^, suggesting that different axes of the UPR^mt^ may regulate distinct aspects of cytosolic proteostasis. In agreement with this possibility, the estrogen receptor alpha (ERα) axis of the UPR^mt^ has been shown to up-regulate the activity of the proteasome^14^, thereby promoting degradation of misfolded and aggregation-prone proteins. Whether the upregulation of proteasome activity by the ERα axis of the UPR^mt^ plays a role in the effect of mitochondrial proteostatic stress on reducing protein aggregates remains unknown.

The ERα is relatively young in evolution, as it first appeared in basal chordates, which are close ancestors of vertebrates^15^. However, some co-activators of the ERα are found earlier in evolution, raising the possibility that an ERα-related mechanism may play a role in the up-regulation of the proteasome by mitochondrial stress in organisms that do not express the ERα, such as yeast. But this possibility remains to be tested.

In vertebrates, the ERα is a major transcription factor that regulates the expression of thousands of genes^16,17^. Its binding to DNA is mediated by two activation function (AF) domains. Binding of estrogen to the AF2 domain induces a conformational change of helix 12 of the ERα promoting its dimerization and exposure of the nuclear localization domain. The AF1 domain, however, is ligand-independent and activated upon phosphorylation by Akt^18^. Importantly for the current study, the activation of the ERα by mitochondrial stress was shown to be dependent on Akt^19^. Genome-wide analysis of the binding of the ERα revealed that several genes are uniquely activated only upon phosphorylation by Akt and not by estrogen alone^20^. Further, binding of the ERα to specific sites in the genome is largely determined by the epigenetic landscape and is highly tissue specific^21^.

The baseline level of proteasome activity is also tissue specific, with the spinal cord among the tissues with the lowest activity^22^. Proteasome activity was reported to show sexual dimorphism, with females having a higher baseline proteasome activity compared to age matched males^22^. This difference between sexes results in significant physiological consequences, as males are more prone to accumulation of ubiquitinated proteins destined for degradation by the proteasome in response to heat shock^22^.

Therefore, in the current study, we tested whether systemic doxycycline administration activates the proteasome through the ERα axis of the UPR^mt^ specifically in tissues prone to proteins aggregation due to low proteasome activity. We also investigated sexual dimorphism in the response to doxycycline.

## Results and discussion

**Figure 1:**
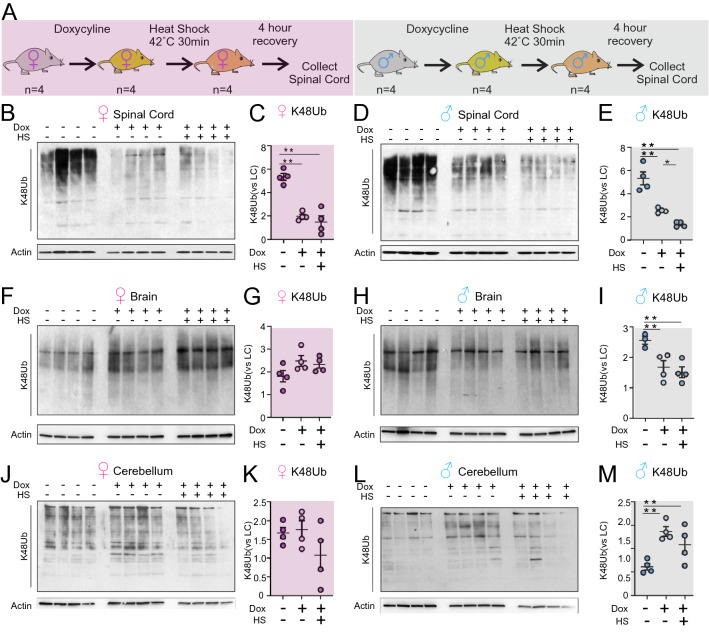
Doxycycline reduces accumulation of CNS K48Ub-linked proteins in a region and sex specific manner. (**A**) Schematic of the experimental design, (**B**) Western blot of K48Ub-linked proteins in the spinal cords of wild-type female mice (n = 4) that were untreated or treated with doxycycline (Dox) alone or with heat shock (HS). Loading was determined using actin. (**C**) Quantification of (**B**). (**D**) Western blot of K48Ub-linked proteins in the spinal cords of wild-type male mice (n = 4) that were untreated or treated with doxycycline (Dox) alone or with heat shock (HS). Loading was determined using actin. (**E**) Quantification of (**D**). (**F**) Western blot of K48Ub-linked proteins in the brain of wild-type female mice (n = 4) that were untreated or treated with doxycycline (Dox) alone or with heat shock (HS). Loading was determined using actin. (**G**) Quantification of (**F**). (**H**) Western blot of K48Ub-linked proteins in the brain of wild-type male mice (n = 4) that were untreated or treated with doxycycline (Dox) alone or with heat shock (HS). Loading was determined using actin. (**I**) Quantification of (**H**). (**J**) Western blot of K48Ub-linked proteins in the cerebellum of wild-type female mice (n = 4) that were untreated or treated with doxycycline (Dox) alone or with heat shock (HS). Loading was determined using actin. (**K**) Quantification of (**J**). (**L**) Western blot of K48Ub-linked proteins in the cerebellum of wild-type male mice (n = 4) that were untreated or treated with doxycycline (Dox) alone or with heat shock (HS). Loading was determined using actin. (**M**) Quantification of L. * or **, indicates *p* < 0.05 or *p* < 0.005 by student’s t-test. For western blots in all panels, samples were run on the same gel in the indicated order with an empty well between groups to facilitate visualization and none of the images were cropped.

The observation that protein aggregates are observed in several neurodegenerative diseases suggest that defect in proteostasis is especially toxic to the nervous system. We hypothesized that inducing UPR^mt^ with a mitochondrial protein translation inhibitor would affect the proteostatic mechanisms of the CNS. In order to test this hypothesis, we first investigated the effects of doxycycline on proteosomal protein degradation in vivo. Female and male mice at 3 months of age were treated with doxycycline in the drinking water for 30 days (Fig. 1A). Following doxycycline treatment, one cohort of mice was exposed to heat shock (40–42 °C) for 30 min and allowed to recover for 4 h (Fig. 1A), while another cohort was maintained at normal temperature. Heat shocking provides additional proteatastic stress by promoting protein misfolding, which leads to increased ubiquitylation of misfolded proteins targeting them for proteasome dependent degradation^23^. Mice were sacrificed and different regions of the central nervous system (forebrain, cerebellum, and spinal cord) were dissected and processed for Western blot analysis using a ubiquitin antibody that specifically recognizes proteins linked to ubiquitin chains formed on lysine 48 of ubiquitin (K48Ub), which target proteins for degradation by the 26S proteasome.

We found that the amount of K48Ub-linked proteins in the spinal cord is drastically reduced in both females and males (Fig. 1B–E) following doxycycline treatment relative to untreated mice. Further, while heat shock was found to significantly increase the accumulation of K48Ub-linked proteins in the spinal cord^22^, the effect of heat shock on accumulation of K48Ub-linked proteins was abolished upon pre-treatment with doxycycline in both sexes (Fig. 1B–E).

Since a proteostatic effect of doxycycline was reported in a model of Alzheimer’s disease^12^, where the forebrain is mostly affected, we next analyzed the brain in our mice cohort. We found that doxycycline significantly reduces K48Ub-linked proteins in the brain of males in presence or absence of heat shock, but not in females (Fig. 1F–I). We also tested the effect of doxycycline in the cerebellum and found that, while it has no effect in females, in males the opposite response was observed, with a trend toward increased accumulation of K48Ub-linked proteins (Fig. 1J–M). These results indicate that the proteostatic effect of doxycycline in the CNS is not only region specific, but also different between sexes.

The tissue and sex specific effect of doxycycline may explain the discrepancy among studies of the impact of doxycycline on the UPR^mt^^8,12,24–26^. Further adding to this discrepancy is the fact that the markers used to monitor the activation of the UPR^mt^ vary widely among studies. We therefore investigated an extended panel of markers of the UPR^mt^ to include markers of the CHOP/ATF4/5, SIRT3, and ERα axes, specifically in the spinal cord, in both males and females^27^. The spinal cord was selected due to the fact that it was found to be among the tissue that shows the lowest proteasome activity^22^.

**Figure 2:**
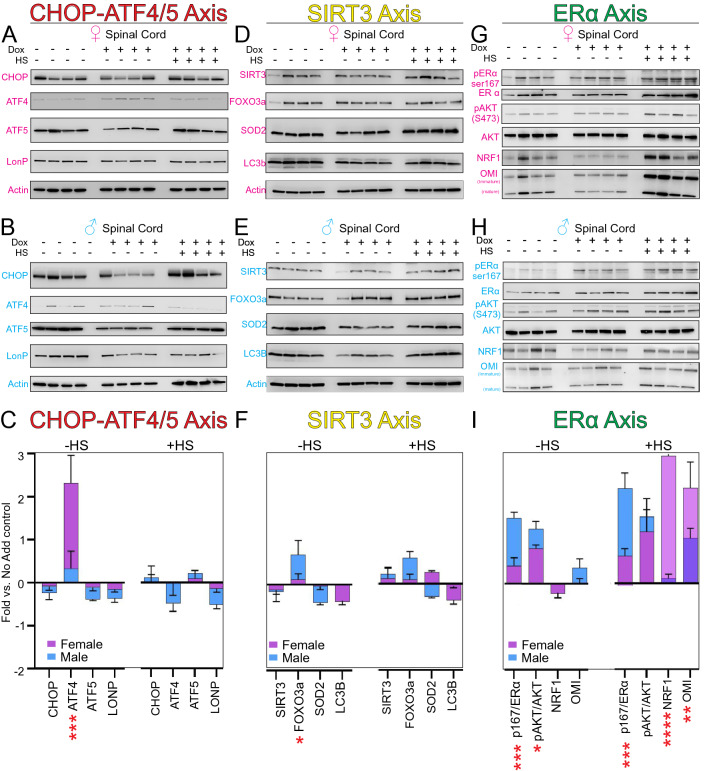
Doxycycline activates the ERα axis of the UPR^mt^ in the spinal cord. (**A**) Western blot of the indicated markers of the CHOP-ATF4/ATF5 axis of the UPR^mt^ in the spinal cord of female mice (n = 4) untreated or treated with doxycycline (Dox) alone or with heat shock (HS). (**B**) Western blot of the indicated markers of the CHOP-ATF4/ATF5 axis of the UPR^mt^ in the spinal cord of male mice (n = 4) untreated or treated with doxycycline (Dox) alone or with heat shock (HS). (**C**) Quantification of (**A**) and (**B**). Values were normalized to actin. Graphs indicate change relative to the untreated control. (**D**) Western blot of the indicated markers of the SIRT3 axis of the UPR^mt^ in the spinal cord of female mice (n = 4) untreated or treated with doxycycline (Dox) alone or with heat shock (HS). (**E**) Western blot of the indicated markers of the SIRT3 axis of the UPR^mt^ in the spinal cord of male mice (n = 4) untreated or treated with doxycycline (Dox) alone or with heat shock (HS). (**F**) Quantification of (**D**) and (**E**). Values were normalized to actin. Graphs indicate change relative to the untreated control. (**G**) Western blot of the indicated markers of the ERα axis of the UPR^mt^ in the spinal cord of female mice (n = 4) untreated or treated with doxycycline (Dox) alone or with heat shock (HS). (**H**) Western blot of the indicated markers of the ERα axis of the UPR^mt^ in the spinal cord of male mice (n = 4) untreated or treated with doxycycline (Dox) alone or with heat shock (HS). (**I**) Quantification of (**G**) and (**H**). Values were normalized to actin. Graphs indicate change relative to the untreated control. * or **, indicates *p* < 0.05 or *p* < 0.005 comparing females to males. For western blots in all panels, samples were run on the same gel in the indicated order with an empty well between groups to facilitate visualization and none of the images were cropped.

For the CHOP-ATF4/5 axis, CHOP, ATF4, ATF5 and LonP were used as markers. We found that with the exception of ATF4 upon doxycycline treatment in female mice only, no other markers were consistently activated with and without heat shock and doxycycline. (Fig. 2A–C). For the SIRT3 axis, SIRT3, Foxo3a, SOD2 and LC3b were used as markers. Similarly, to the CHOP-ATF/5 axis, doxycycline with or without heat shock also failed to activate the SIRT3 axis of the UPR^mt^ (Fig. 2D–F). For the ERα axis, phospho- ERα, phospho-Akt, NRF1 and Omi were used as markers. We found a significant activation of 2 out of 4 markers (phospho- ERα, phospho-Akt) was observed in both males and females treated with doxycycline alone, and activation of all 4 makers of this axis was observed when doxycycline was combined with heat shock (Fig. 2G–I). These results suggest that the ERα-mediated activation of the proteasome may play a role in the reduction in K48Ub-linked proteins upon treatment with doxycycline, with and without heat shock. Further, consistent with the observation that activation of the proteasome by ERα requires phosphorylation by Akt^19^, we found that phospho-Akt was significantly increased, but interestingly more so in males (Fig. 2G–I). Why doxycycline and heat shock result in the preferential activation of the ERα axis of the UPR^mt^ and not the others is unclear but it suggests that proteotoxic stress is a more potent activator of this axis. Logically since this is the axis shown to promote proteasome activation, its activation appears as the most adapted response to resolve this particular stress.

**Figure 3:**
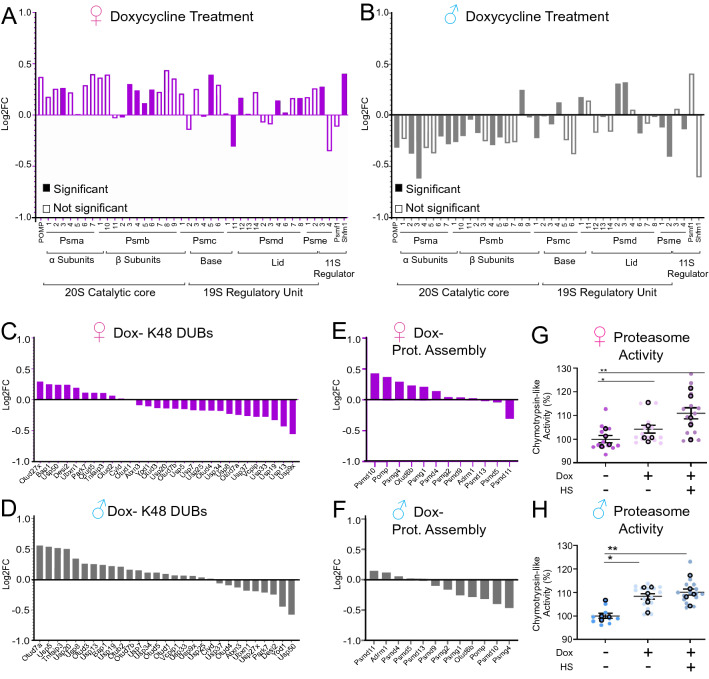
Doxycycline activates the transcription of proteasome genes in females. (**A**, **B**) Analysis of the relative expression of proteasome genes of the 20S, 19S and11S in the spinal cord of females (n = 4) (A) and males (n = 4) (**B**) following treatment with doxycycline. Significance is indicated if *p* < 0.05 by student’s t-test. (**C**, **D**) Analysis of the relative expression of deubiquitinating enzymes genes in the spinal cord of females (n = 4) (**C**) and males (n = 4) (**D**) following treatment with doxycycline. (**E**–**F**) Analysis of differential gene expression for proteasome assembly genes in the spinal cord of females (n = 4) (**E**) and males (n = 4) (**F**) following treatment with doxycycline. (**G**, **H**) Proteasome activity with and without doxycycline alone or with heat shock (HS) in the spinal cord of females (n = 4) (**G**) and males (n = 4) (**H**). * or **, indicates *p* < 0.05 or *p* < 0.005 by student’s t-test.

To directly address the effect of doxycycline on the proteasome, we performed four analyses. First, we interrogated RNAseq data for the expression of proteasome subunits, including all genes of the 20S catalytic core as well as the 19S and 11S regulatory cores. We found that doxycycline treatment increased the expression of most proteasome subunits in females, but not males (Fig. 3A,B). Second, we analyzed the expression of K48-Ub de-ubiquitinating enzymes (DUBs) and found, again, an opposite trend between males and females, with females mainly showing a down-regulation of DUBs, and males mainly an up-regulation of DUBs, following doxycycline treatment (Fig. 3C,D). Third, we analyzed the expression of genes involved in proteasome assembly and found an up-regulation in females, but a downregulation in males (Fig. 3E,F). Fourth, we measured the activity of the 20S proteasome in spinal cord lysates using a commercially available fluorogenic substrate. Interestingly, increased proteasome activity was observed in both sexes following doxycycline treatment (Fig. 3G,H). These findings suggest that doxycycline increases proteasome activity and decreases UbK48-linked proteins in both sexes, but this effect occurs through distinct mechanisms. In females, but not in males, the increased proteasome activity appears to be mainly due to increased transcription of proteasome subunits and assembly factors. While potential differences in the permeability of the brain blood barrier to doxycycline cannot be rule out, since we found a similar effect on proteasome activity and K48Ub-linked proteins accumulation in both sexes, these results suggest that the concentration of doxycycline to reach the brain is similar in both sexes.

**Figure 4:**
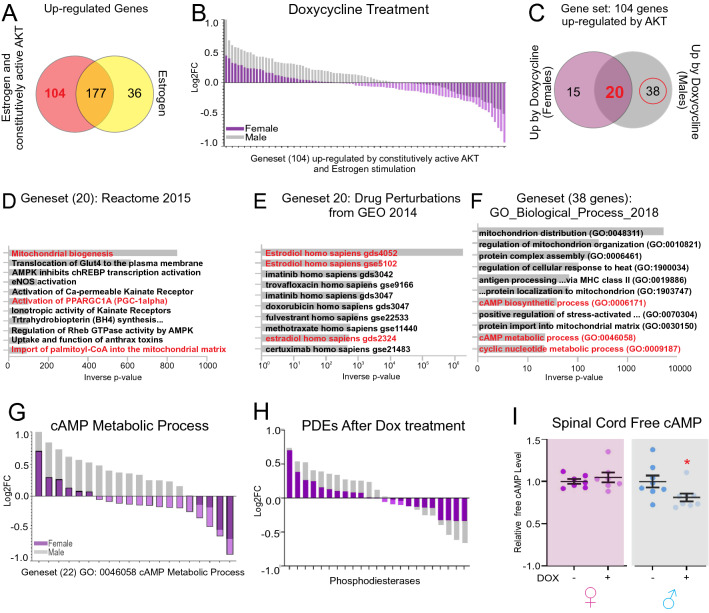
Doxycycline modulates cAMP related processes in males but not female mice. (**A**) Analysis of upregulated genes reported by Bhat-Nakshatri et al.^20^. (**B**) Differential gene expression of genes in the gene set identified in panel (**A**) (104 genes) following treatment with doxycycline. (**C**) Analysis of the genes upregulated by doxycycline treatment in the gene set identified in panel (**A**). (**D**) Reactome analysis of the 20 genes found in panel (**C**) that were commonly up-regulated by females and males following doxycycline treatment. (**E**) Drug perturbation signature analysis of the 20 genes found in panel C that were commonly up-regulated by females and males following doxycycline treatment. (**F**) Gene Ontology analysis of the 38 genes identified in panel (**C**) that were uniquely up-regulated by doxycycline in male mice. (**G**) Differential gene expression of genes in GO:0046058 in female and male mice following doxycycline treatment. (**H**) Differential gene expression of genes encoding phosphodiesterases in female and male mice following doxycycline treatment. (**I**) ELISA detecting free cAMP in spinal cord lysates from female (n = 4) or male (n = 4) with or without doxycycline treatment (n = 4 mice per group with 2 technical replicates per animal). *p < 0.05 by two-tail student’s t-test.

Since the increase in phosphorylation of both Akt and ERα was more prominent in males following doxycycline treatment (Fig. 2H), and because phosphorylation of the ERα by Akt alters its transcriptional pattern, we next focused on ERα driven genes that are regulated by Akt. Using a publicly available dataset^20^, we interrogated genes regulated by ERα and Akt by comparing genes upregulated by estrogen alone or by estrogen plus constitutively active Akt. We found that 104 genes are uniquely up-regulated by constitutively active Akt in this model (Fig. 4A). The expression of these 104 genes was then analyzed in our cohort of males and females following doxycycline treatment. We found that more genes are upregulated in males and to a greater extent (Fig. 4B). Upon further analysis, we found that 20 of the 104 genes are up-regulated in both males and females (Fig. 4C) and functional clustering of these 20 genes related to promoting mitochondria biogenesis and function (Fig. 4D) and estrogen signaling (Fig. 4E). We then performed the converse analysis and focused on 38 genes that were uniquely upregulated in males following doxycycline treatment. This analysis identified cAMP metabolic process as one of the most significant processes up-regulated by doxycycline specifically in males (Fig. 4F,G). This observation is of interest since cAMP signaling has been associated with regulation of proteasome activity in muscle and liver^28–30^. Increased cAMP levels as a result of growth factor stimulation or fasting have been reported to lead to increased proteasome subunit phosphorylation and subsequent increased proteasome activity mediated by PKA in muscle and liver^31^, while other groups have observed that increased cAMP levels as a result of inhibiting cAMP-phosphodiesterase activity with Isobutylmethylxanthine (IBMX) suppressed proteasome activity^30,32^. We therefore analyzed potential changes in phosphodiesterase expression and found that males increased PDE expression more than females following doxycycline treatment (Fig. 4H). This observation suggests that males may have decreased free cAMP levels due to increased PDE expression following doxycycline treatment. We therefore measured cAMP levels and found a decrease in free cAMP only in males following doxycycline treatment (Fig. 4I). Taken together our findings would suggest that male spinal cord responds to doxycycline similarly to when PDE activity is affected leading to increase proteasome activity.

**Figure 5:**
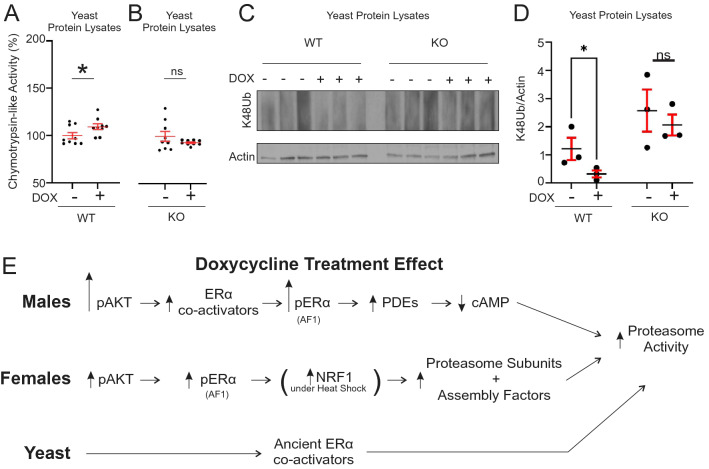
Doxycycline activates the proteasome in yeast in a Mms19 dependent manner. (**A**) Activity of the proteasome in wild-type yeast cells with and without doxycycline (n = 3 biological replicates with 2–3 technical replicates per culture) after 90 min of treatment values were normalized to the untreated control in both groups. (**B**) Activity of the proteasome in Mms19 knockout yeast cells (mms19Δ) with and without doxycycline. (n = 3 biological replicates with 2–3 technical replicates per culture). (**C**) Western blot of K48Ub-linked proteins in wild-type and mms19Δ yeast cells with and without doxycycline (n = 3 biological replicates). The samples were run on the same gel in the indicated order. (**D**) Quantification of (**C**). (**E**) Model of the proposed differential route of activation of the proteasome in yeast or male and female spinal cords. * or **, indicates *p* < 0.05 or *p* < 0.005 by student’s t-test.

Our results suggest that the activation of the ERα axis of the UPR^mt^ may be responsible for the increase in proteasome activity upon treatment with doxycycline, and therefore may explain how doxycycline leads to a reduction in protein aggregates associated with diseases such as Alzheimer disease^12^. However, some of the studies reporting increased proteasome activity upon mitochondrial stress were conducted in organisms that do not express the ERα, such as yeast^7^. We therefore tested if doxycycline also up-regulates the activity of the proteasome in the fission yeast *S. Pombe* and found a significant increase of proteasome activity (Fig. 5A). Since ERα first appeared in basal chordates (cephalochordates: amphioxus), which are close ancestors of vertebrates^15^, we reasoned that some co-activators of the ERα may carry an ancestral function of ERα. We therefore searched for ERα co-activators that are expressed in fission yeast. We found Mms19, which is of particular interest as it is expressed in yeast, translocates from the mitochondria to the nucleus and was shown to act as an AF1 co-activator of ERα^33,34^. To test the potential role of Mms19 on the activation of the proteasome by doxycycline, we obtained a MMS19 knockout yeast strain (mms19Δ) and treated these cells with doxycycline. We found that genetic deletion of Mms19 abolishes the activation of the proteasome by doxycycline after 90 min of treatment (Fig. 5B). We then analyzed the level of K48Ub-linked proteins in wild-type and mms19Δ yeast cells in response to doxycycline and found that, while doxycycline decreases their accumulation in wild-type cells, it does not significantly reduce their levels in the mms19Δ cells (Fig. 5C,D). This result suggests that prior to the appearance of the ERα in evolution, the activation of the proteasome upon mitochondrial stress may have been carried by Mms19 (Fig. 5E). Since the appearance of the ERα in vertebrates, however, the signaling between mitochondrial stress and the proteasome has significantly diversified and become not only tissue specific, but also sex-specific (Fig. 5E).

Mitochondrial stress was also been reported to decrease proteasome activity and promote proteasome disassembly^5^. One potential explanation for this discrepancy is that the effect on the proteasome may be relative to the type, extent and duration of mitochondrial stress since this study was performed under acute mitochondrial stress, while doxycycline represents a milder level of mitochondrial stress. Our data suggest that the mild stress induced by doxycycline promotes mitohormesis, a mechanism by which moderate mitochondrial stress confers a beneficial effect and resistance to subsequent exposure to stress^35–37^.

Proteostasis dysfunction is a unifying theme across neurodegenerative diseases. One central problem in developing interventions against such diseases is that irreversible damage has already taken place upon manifestation of symptoms. The findings presented in the current study suggest that doxycycline, a drug already used widely and well-tolerated clinically, may be a novel therapeutic in the prevention setting, by promoting proteasome activity and delaying proteostasis defects in tissues prone to pathological protein aggregation. In agreement with this possibility, doxycycline was previously shown to reduce β-amyloid aggregates^12^. Here, to further assess the potential therapeutic effects of doxycycline in neurodegeneration, we tested doxycycline effects in a mouse model of familial amyotrophic lateral sclerosis (ALS), the SOD1-G93A mouse model, where the spinal cord accumulates pathological protein aggregates. In female SOD1-G93A mice, we found that doxycycline increased proteasome activity and reduced accumulation of K48Ub-linked proteins in the spinal cord (Suppl. Fig. 1), suggesting that doxycycline may play a neuroprotective role. Lastly, the potential clinical use of doxycycline is also supported by a recent study indicating that it may be effective in mitochondrial diseases^38^. Taken together, these findings suggest that modulating UPR^mt^-induced proteostasis in the CNS with doxycycline could be a viable therapeutic strategy in neurodegenerative diseases and other proteopathic diseases. However, our results also warn that the effect of doxycycline must be studied in a tissue and sex specific manner. Why doxycycline affects the accumulation of K48Ub-linked proteins in the spinal cord but does not have the same effect in the cerebellum remains unclear. One possibility is that the expression of the ERα varies between tissues and cell types. Therefore, more studies will be required to fully understand the tissue specificity of doxycycline in the future.

## Material and methods

### Mice

All experiments were approved by the Mt. Sinai Institutional Animal Care and Use Committee (IACUC) and performed according to the principles of laboratory animal care outlined in NIH publication No. 86-23, revised 1985 edition. All experiments in mice were conducted in compliance with the ARRIVE guidelines. Mice were either wild-type FVBN mice harvested between 3 and 5 months of age or B6SJL-TG_SOD1*G93A)1Gur/J (available from the Jackson Laboratory). All animals were maintained according to IACUC approved methods. SOD1-G93A mice were sacrificed in average at 69 days of age, before observable symptoms manifested. All collected tissue was immediately frozen on dry ice and stored for later analysis.

### Doxycycline treatment

FVBN mice were administered doxycycline hyclate in their drinking water (1.5 g/L) for 30 days ad libitum, with fresh suspension every 3–4 days. SOD1-G93A mice were administered doxycycline by IP injections daily for 5 days.

### Heat shock

Heat shock was performed as previously described^22,39^. Briefly, four mice were kept in a ventilated box maintained at 41–43 °C for 20 min using a standard heating lamp, with temperature monitoring accomplished by a suspended alcohol thermometer in the center of the box. Tissues were harvested 4 h after following treatment. Body temperatures were monitored prior and after heat shock using rectal thermometers and showed resting temperatures of 37.24 ± 0.26 °C (n = 4) and of 39.53 +  − 0.41 °C (n = 4) after heat shock.

### Tissue collection, soluble and insoluble fractions preparation

Tissue collection and processing was performed as previously described^22^. Briefly, frozen tissue powders were generated using pestle and mortar on dry ice. Tissues were lysed in NP40 lysis buffer (50 mM Tris, 250 mM NaCl, 5 mM EDTA, 0.5% NP-40, 50 mM NaF, 1 mM DTT plus, PR-619 (Selleck Chemicals) (20 µM), and protease inhibitors) using a 1:4 volume to volume ratio of tissue powder to lysis buffer. All samples were subjected to probe sonication (fisher scientific FB505) with 2–3 rounds of 1 s intervals at 20% amplitude on ice. Protein lysates were centrifuge at 20,800 g at 4 °C for 20 min. The top layer (soluble fraction) was transferred to a new tube. The remaining pellet was termed “the insoluble fraction”. Pellet volumes were estimated by pipette and an equivalent volume of TEN buffer (10 mM Tris-HcCl, 1 mM EDTA, 100 mM NaCl plus protease inhibitors) was added to make a 1:1 pellet:TEN buffer suspension. Samples were then sonicated twice with 1 s intervals at 20% amplitude on ice. This suspension was then quantified by Bio-Rad Protein Assay Dye method (cat. No. 500–0006). 15 µg of this suspension was adjusted to 1ug/uL, then added to an equal volume of 5 × loading buffer (1 M tris–HCl, 25% Glycerol, 2% SDS, 5% 2-mercaptoethanol, 0.1% bromophenol blue) and heated to 95 °C for 5 min before being separated by electrophoresis.

### Western blot blotting

Western blot blot was performed as described^22^. Analysis was performed using the Bio-Rad Criterion Cell Midi blot system. Proteins were collected as described above and quantified using the Bio-Rad Protein Assay Dye method (cat. No. 500-0006) before being separated on a 10% or 4–20% gradient Precast Midi (Bio-Rad, cat. No. 5671094) polyacrylamide gel and transferred to a nitrocellulose blotting membrane (GE Healthcare Life Sciences). Following transfer, membranes were blocked in 5% non-fat dry milk suspended in Tris-buffered saline plus 0.1% Tween 20 (TBST) for 45–60 min at room temperature. With the exception of membranes probed with K48Ub (EMD-Millilore, cat. No. 05-1307), which requires the entire membrane, the membranes were then cut around the appropriate molecular weight prior to hybridization with primary antibodies against Omi/HtrA2 (Biovision cat No. 3497-100), Actin (Santa Cruz Biotechnology, cat. No. sc-47778), K48Ub (EMD-Millilore, cat. No. 05-1307), Omi/HtrA2 (Biovision cat No. 3497-100), Actin (Santa Cruz Biotechnology, cat. No. sc-47778), pERα (S167) (Abcam cat. No. ab31478), ERα (Santa Cruz Biotechnology cat. No. sc-8005), AKT (cell signaling technology, cat. No. 9272), pAKT (S473) (cell signaling technology, cat. No. #4051S), NRF1 (Abcam, cat. No. ab55744), ATF4 (Proteintech, cat. No. 60035-1-Ig), ATF5 (Abcam, cat. No. ab184923), FOXO3a (75D8) Rabbit mAb (Cell Signaling cat. No. 2493S), SOD2 (Millipore, cat. No. 06-984), LC3B (Cell Signalling, cat. No. 2775S), SIRT3 (Abcam, cat. No. ab264041, CHOP (Cell Signalling (L63F7) cat. No. 2895S), LONP1/Lon (Abcam, cat. No. ab103809). All primary antibodies were suspended in 2.5% milk TBST, except for antibodies directed again phosphorylated residues, which were suspending in 2.5% BSA TBST. After 3 washes in TBST, blots were probed with horseradish peroxidase conjugated anti-mouse (Kindle Biosciences, cat. No. 1005) or anti-rabbit (Kindle Biosciences, cat No. R1006) antibodies and detected using enhanced chemiluminescence (Millipore or Kindle Biosciences). Images of developed blots were captured either using film and then digitized with a flatbed scanner, or captured using the KwikQuant Imager system (Kindle Biosciences, cat. No. D1001). Bands quantification were performed in ImageJ using the built in densitometry tools.

### Proteasome fluorescence assay

Proteasome activity was assessed as previously described^22,40^. Briefly, 20 µg of tissue protein lysates were added to proteasome activity assay buffer (50 mM Tris–HCl, pH 7.5) along with 10 µM of chymotrypsin (Suc-LLVY-AMC) (Calbiochem, cat. No. 539142) fluorogenic proteasome substrate. Reactions were incubated at 37 °C protected from light for 3 h. Following incubation, samples were transferred to black walled 96 well plates (Greiner Bio-One, cat No. 655097). Release of free 7-amino-4-methyl-coumarin (AMC) was determined using a SpectraMax M5e microplate reader (Molecular Devices) with excitation at 380 nm and emission recorded at 460 nm. Reported values were normalized to the no treatment control in each group.

### RNA sequencing and analyses

Flash frozen spinal cord tissue (n = 4 Female, n = 4 male) was sent to Genewiz (South Plainfield, NJ) for next generation RNA sequencing (with two technical replicates per animal) and previously described^41^. Raw .fastq files were supplied and are available on the BioJupies cloud^42^. (https://maayanlab.cloud/biojupies/analyze/tools?uid=ETnRFsqk9Be).

Analysis of RNAseq fastq files was performed using the BioJupies suite of tools for alignment, annotation, and differential expression analysis of our treatment groups. Gene ontology enrichment analysis was performed using the ontology enrichment analysis tool for biological processes provided by The Gene Ontology Consortium or the suite of analysis tools provided through Enrichr. Genesets (GSEA Gene Sets: BHAT_ESR1_TARGETS_VIA_AKT1_UP and BHAT_ESR1_TARGETS_NOT_VIA_AKT1_UP) identified by Bhat-Nakshatri et al.^20^ were used to identify ERS1 target genes that are up-regulated by AKT1.

### Fission yeast methods

Multiple independent isogenic wild-type and *mms19∆* cultures were grown to mid-logarithmic phase in YES medium^43^ and treated with 5 µM doxycycline or vehicle (DMSO) for 90 min. Cells were harvested by centrifugation and snap-frozen in liquid nitrogen. Cell extracts were prepared in NP40 lysis buffer following cell disruption in a mini-bead beater (Biospec). Extracts were clarified at 16,100 g at 4 °C for 10 min, and processed for western blotting and proteasome assays as described below.

### Statistics

Data on graphs is represented as the mean ± SEM. Student’s T-test or ANOVA with a Bonferroni multiple comparison post-test was used to ascertain a significant difference in mean when comparing two groups.

## Supplementary Information

Supplementary Information 1.

Supplementary Information 2.

## References

[1] Baker BM, Nargund AM, Sun T, Haynes CM (2012). Protective coupling of mitochondrial function and protein synthesis via the eIF2alpha kinase GCN-2. PLoS Genet..

[2] Rainbolt TK, Atanassova N, Genereux JC, Wiseman RL (2013). Stress-regulated translational attenuation adapts mitochondrial protein import through Tim17A degradation. Cell Metab..

[3] Labbadia J (2017). Mitochondrial stress restores the heat shock response and prevents proteostasis collapse during aging. Cell Rep..

[4] Kim HE (2016). Lipid biosynthesis coordinates a mitochondrial-to-cytosolic stress response. Cell.

[5] Livnat-Levanon N (2014). Reversible 26S proteasome disassembly upon mitochondrial stress. Cell Rep..

[6] Segref A (2014). Pathogenesis of human mitochondrial diseases is modulated by reduced activity of the ubiquitin/proteasome system. Cell Metab..

[7] Wrobel L (2015). Mistargeted mitochondrial proteins activate a proteostatic response in the cytosol. Nature.

[8] Houtkooper RH (2013). Mitonuclear protein imbalance as a conserved longevity mechanism. Nature.

[9] Atakan HB, Hof KS, Cornaglia M, Auwerx J, Gijs MAM (2019). The detection of early epigenetic inheritance of mitochondrial stress in *C. elegans* with a microfluidic phenotyping platform. Sci. Rep..

[10] Chatzispyrou IA, Held NM, Mouchiroud L, Auwerx J, Houtkooper RH (2015). Tetracycline antibiotics impair mitochondrial function and its experimental use confounds research. Cancer Res..

[11] Moullan N (2015). Tetracyclines disturb mitochondrial function across eukaryotic models: A call for caution in biomedical research. Cell Rep..

[12] Sorrentino V (2017). Enhancing mitochondrial proteostasis reduces amyloid-beta proteotoxicity. Nature.

[13] Anderson NS, Haynes CM (2020). Folding the mitochondrial UPR into the integrated stress response. Trends Cell Biol..

[14] Radke S (2008). Mitochondrial protein quality control by the proteasome involves ubiquitination and the protease Omi. J. Biol. Chem..

[15] Baker ME (2019). Steroid receptors and vertebrate evolution. Mol. Cell Endocrinol..

[16] Williams C, Edvardsson K, Lewandowski SA, Strom A, Gustafsson JA (2008). A genome-wide study of the repressive effects of estrogen receptor beta on estrogen receptor alpha signaling in breast cancer cells. Oncogene.

[17] McBryan J, Howlin J, Kenny PA, Shioda T, Martin F (2007). ERalpha-CITED1 co-regulated genes expressed during pubertal mammary gland development: Implications for breast cancer prognosis. Oncogene.

[18] Cagnet S (2018). Oestrogen receptor alpha AF-1 and AF-2 domains have cell population-specific functions in the mammary epithelium. Nat. Commun..

[19] Papa L, Germain D (2011). Estrogen receptor mediates a distinct mitochondrial unfolded protein response. J. Cell Sci..

[20] Bhat-Nakshatri P (2008). AKT alters genome-wide estrogen receptor alpha binding and impacts estrogen signaling in breast cancer. Mol. Cell Biol..

[21] Achinger-Kawecka J (2020). Epigenetic reprogramming at estrogen-receptor binding sites alters 3D chromatin landscape in endocrine-resistant breast cancer. Nat. Commun..

[22] Jenkins EC (2020). Proteasome mapping reveals sexual dimorphism in tissue-specific sensitivity to protein aggregations. EMBO Rep..

[23] Fang NN (2014). Rsp5/Nedd4 is the main ubiquitin ligase that targets cytosolic misfolded proteins following heat stress. Nat. Cell Biol..

[24] Wang YT (2019). Cardioprotection by the mitochondrial unfolded protein response requires ATF5. Am. J. Physiol. Heart Circ. Physiol..

[25] Ozkurede U, Miller RA (2019). Improved mitochondrial stress response in long-lived Snell dwarf mice. Aging Cell.

[26] Michel S, Canonne M, Arnould T, Renard P (2015). Inhibition of mitochondrial genome expression triggers the activation of CHOP-10 by a cell signaling dependent on the integrated stress response but not the mitochondrial unfolded protein response. Mitochondrion.

[27] Kenny TC, Germain D (2017). From discovery of the CHOP axis and targeting ClpP to the identification of additional axes of the UPRmt driven by the estrogen receptor and SIRT3. J. Bioenerg. Biomembr..

[28] Silveira WA (2014). Activating cAMP/PKA signaling in skeletal muscle suppresses the ubiquitin-proteasome-dependent proteolysis: implications for sympathetic regulation. J. Appl. Physiol..

[29] Gonçalves DAP (2009). Mechanisms involved in 3′,5′-cyclic adenosine monophosphate-mediated inhibition of the ubiquitin-proteasome system in skeletal muscle. Endocrinology.

[30] Lira EC (2011). Phosphodiesterase-4 inhibition reduces proteolysis and atrogenes expression in rat skeletal muscles. Muscle Nerve.

[31] VerPlank JJS, Lokireddy S, Zhao J, Goldberg AL (2019). 26S Proteasomes are rapidly activated by diverse hormones and physiological states that raise cAMP and cause Rpn6 phosphorylation. Proc. Natl. Acad. Sci..

[32] Goncalves DA (2012). Clenbuterol suppresses proteasomal and lysosomal proteolysis and atrophy-related genes in denervated rat soleus muscles independently of Akt. Am. J. Physiol. Endocrinol. Metab..

[33] Wu X, Li H, Chen JD (2001). The human homologue of the yeast DNA repair and TFIIH regulator MMS19 is an AF-1-specific coactivator of estrogen receptor. J. Biol. Chem..

[34] Wu R (2018). MMS19 localizes to mitochondria and protects the mitochondrial genome from oxidative damage. Biochem. Cell Biol..

[35] Kenny TC, Gomez ML, Germain D (2019). Mitohormesis, UPR(mt), and the complexity of mitochondrial DNA landscapes in cancer. Cancer Res..

[36] Yun J, Finkel T (2014). Mitohormesis. Cell Metab..

[37] Palmeira CM (2019). Mitohormesis and metabolic health: The interplay between ROS, cAMP and sirtuins. Free Radic. Biol. Med..

[38] Perry EA (2021). Tetracyclines promote survival and fitness in mitochondrial disease models. Nat. Metab..

[39] Vallanat B (2010). Analysis of the heat shock response in mouse liver reveals transcriptional dependence on the nuclear receptor peroxisome proliferator-activated receptor alpha (PPARalpha). BMC Genomics.

[40] Riar AK (2017). Sex specific activation of the ERalpha axis of the mitochondrial UPR (UPRmt) in the G93A-SOD1 mouse model of familial ALS. Hum. Mol. Genet..

[41] Jenkins EC (2021). Raloxifene is a female-specific proteostasis therapeutic in the spinal cord. Endocrinology.

[42] Torre D, Lachmann A, Ma’ayan A (2018). BioJupies: Automated generation of interactive notebooks for RNA-Seq data analysis in the cloud. Cell Syst..

[43] Moreno S, Klar A, Nurse P (1991). Molecular genetic analysis of fission yeast Schizosaccharomyces pombe. Methods Enzymol..

