# Supplementary material for: Doxycycline promotes proteasome fitness in the central nervous system
Source: Sci Rep. 2021 Aug 20;11:17003. doi: 10.1038/s41598-021-96540-z (PMC8379233; doi:10.1038/s41598-021-96540-z)
Supplement: Supplementary file 1 — Supplementary Information 1. [file 41598_2021_96540_MOESM1_ESM.pdf]

Supplementary figure 1

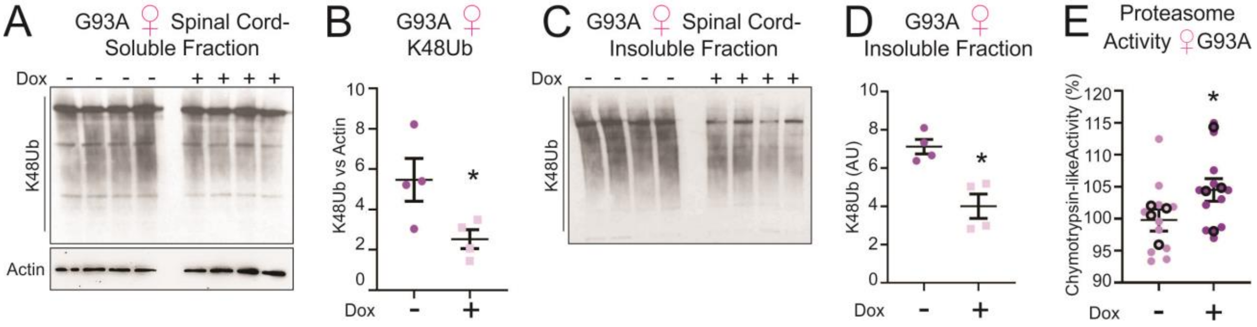

Supplementary Figure 1: **Doxycycline reduces accumulation of Ub-K48-linked proteins in the spinal cord of the SOD1-G93A model of ALS.** A) Western blot of Ub-K48-linked proteins in the soluble fraction of the spinal cords of SOD1-G39A female mice (n=4) that were untreated or treated with doxycycline (Dox). Loading was determined using actin. B) Quantification of A. C) Western blot of Ub-K48-linked proteins in the insoluble fraction of the spinal cords of SOD1-G93A female mice (n=4) that were untreated or treated with doxycycline (Dox). Loading was determined using actin. D) Quantification of C. E) Proteasome activity with and without doxycycline in the spinal cord of SOD1-G93A females (n=4).
