# Supplementary material for: Doxycycline promotes proteasome fitness in the central nervous system
Source: Sci Rep. 2021 Aug 20;11:17003. doi: 10.1038/s41598-021-96540-z (PMC8379233; doi:10.1038/s41598-021-96540-z)

Figure 1 B

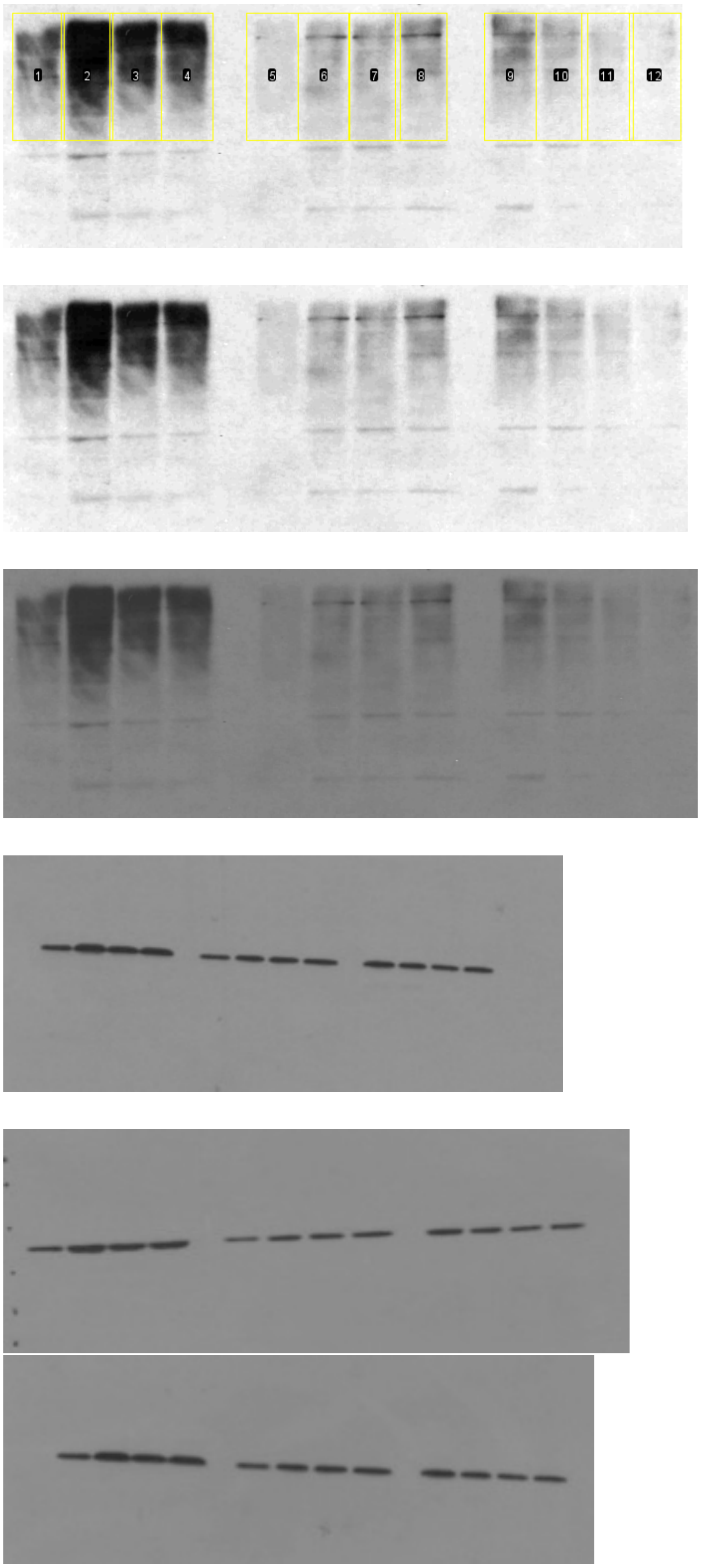

Figure 1 B

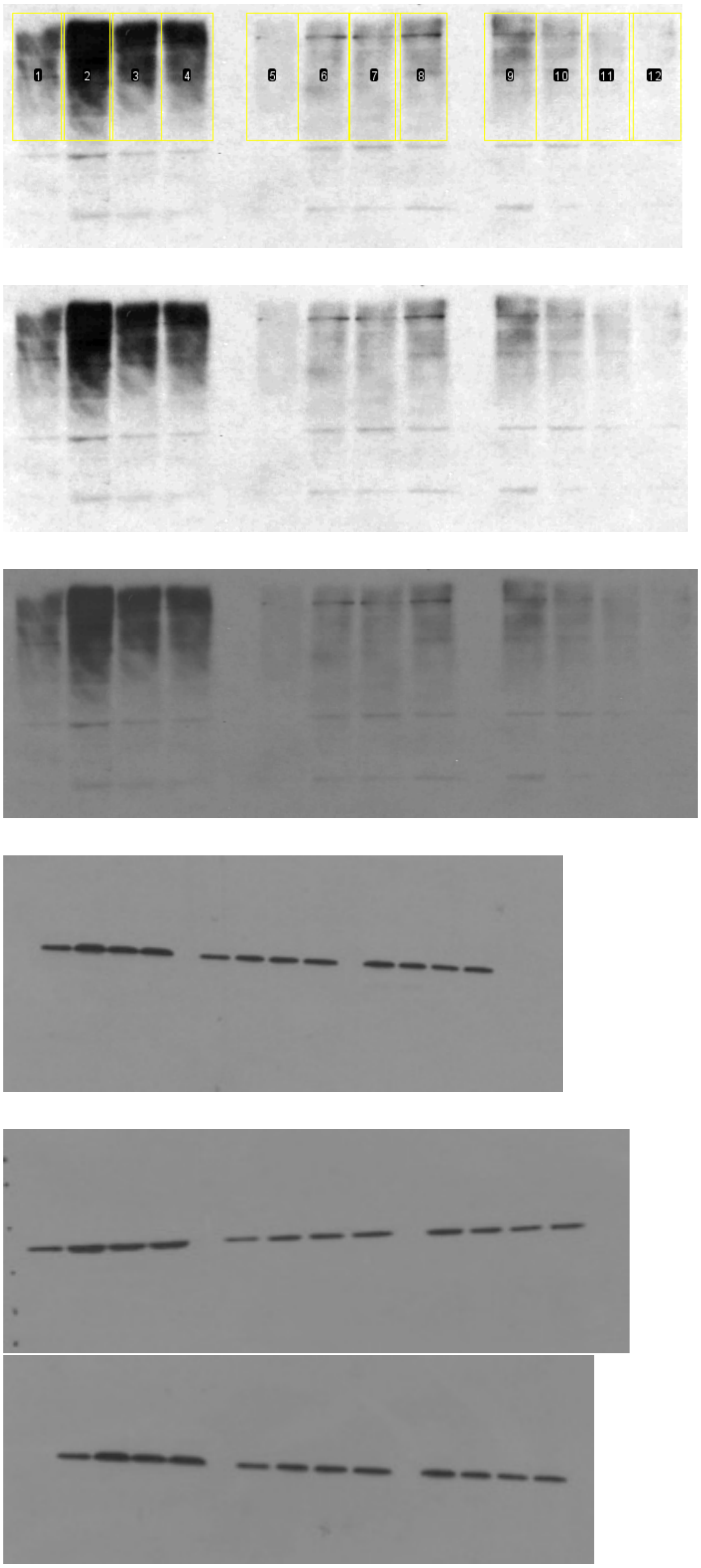

Figure 1D

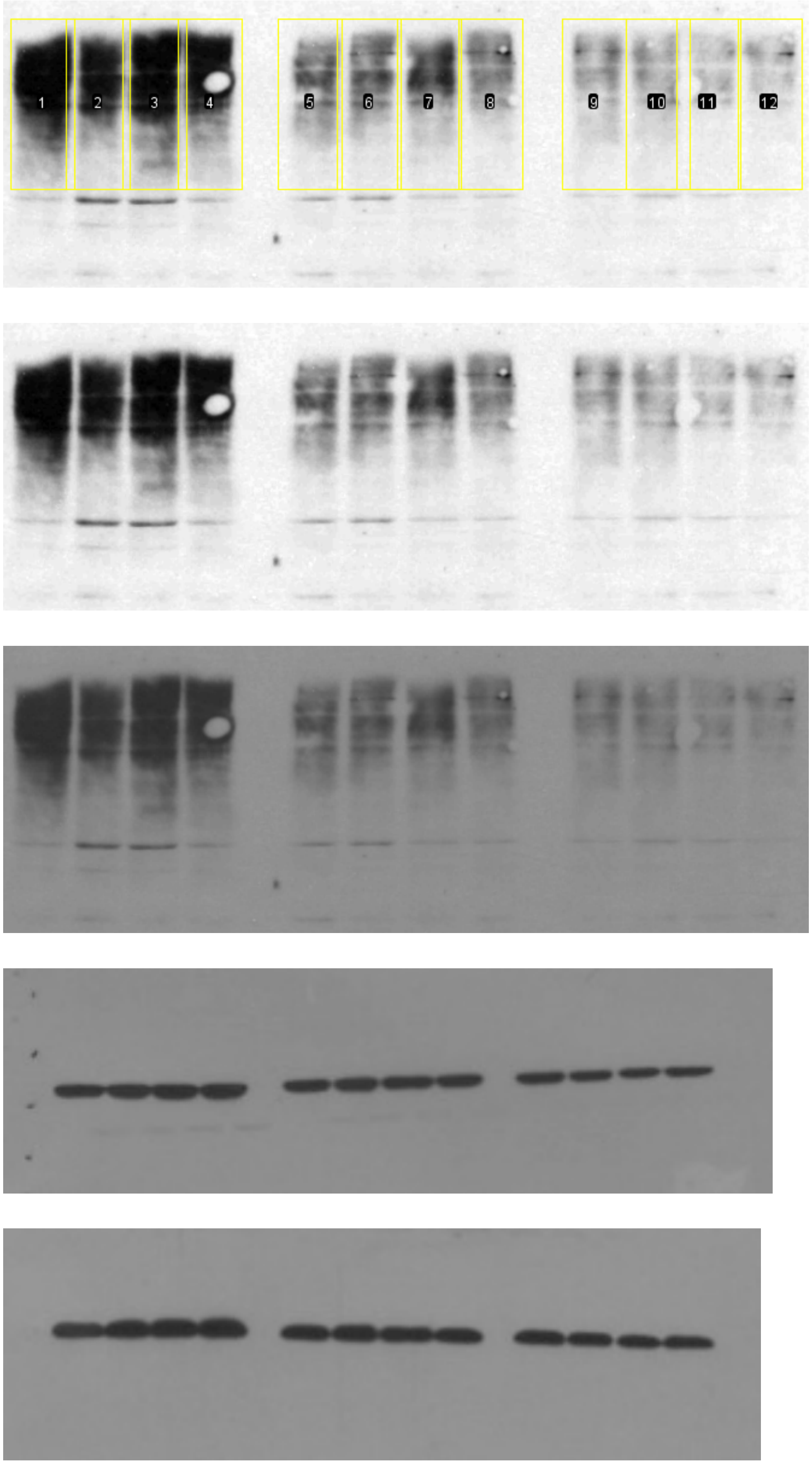

Figure 1F

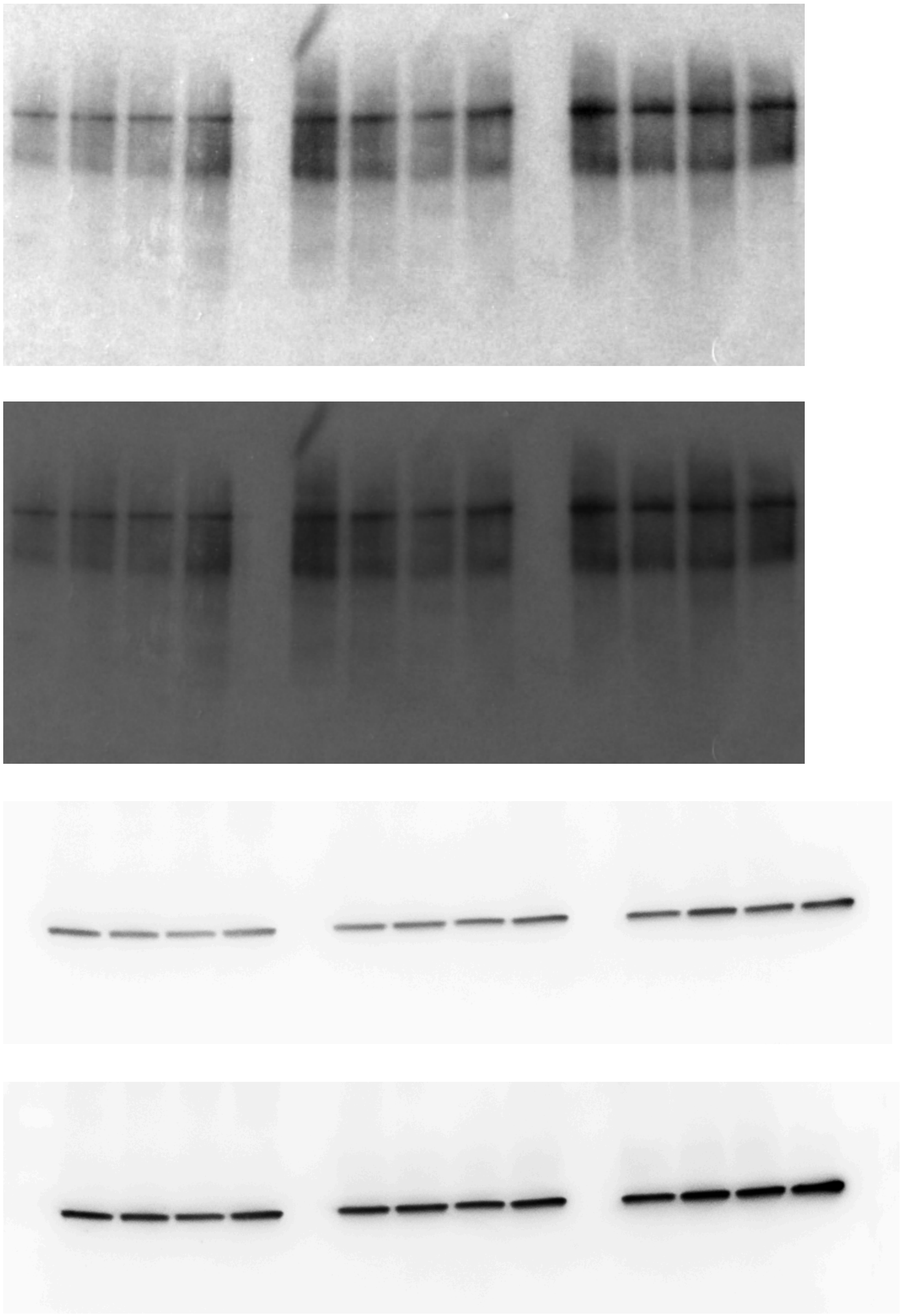

Figure 1H

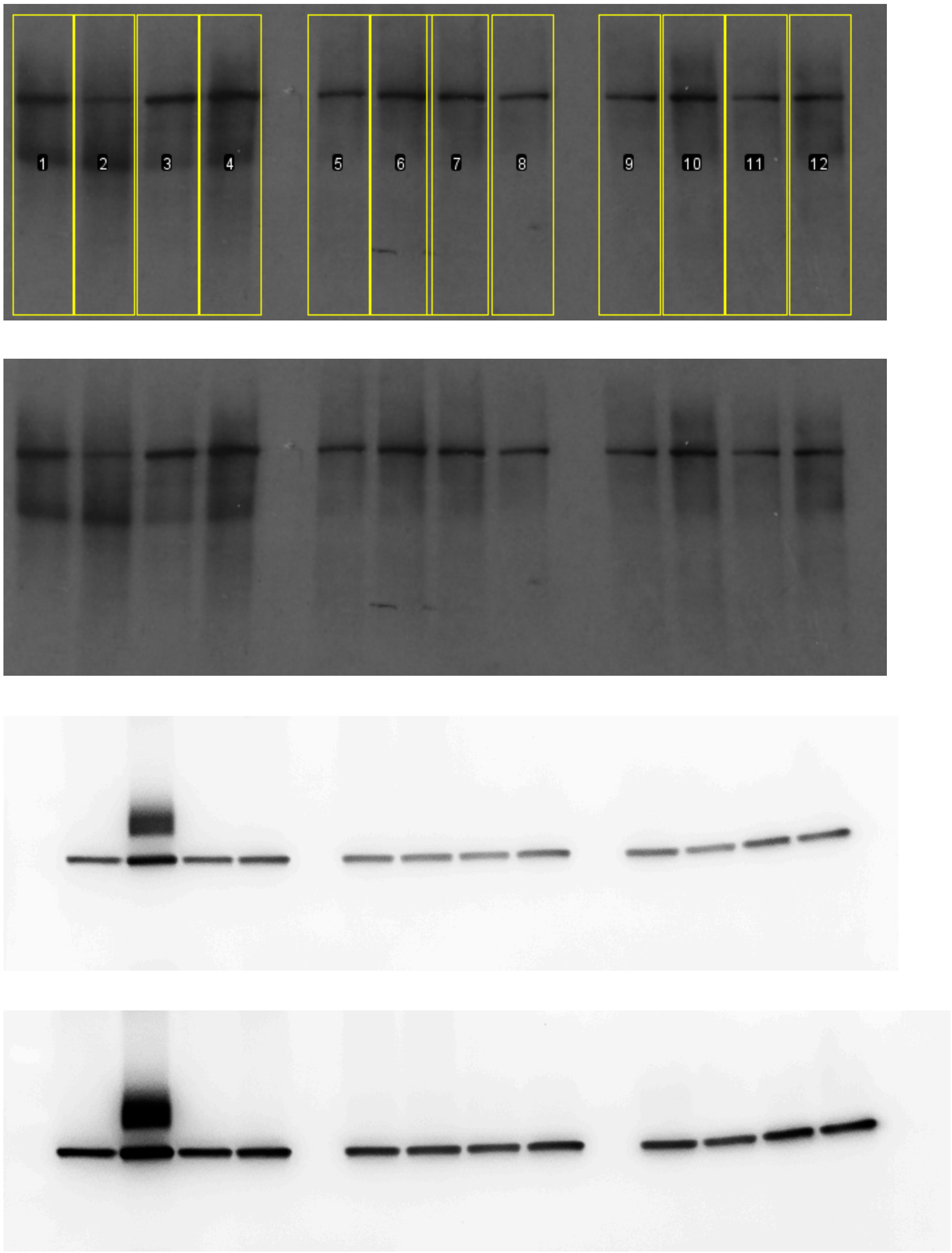

Figure 1J

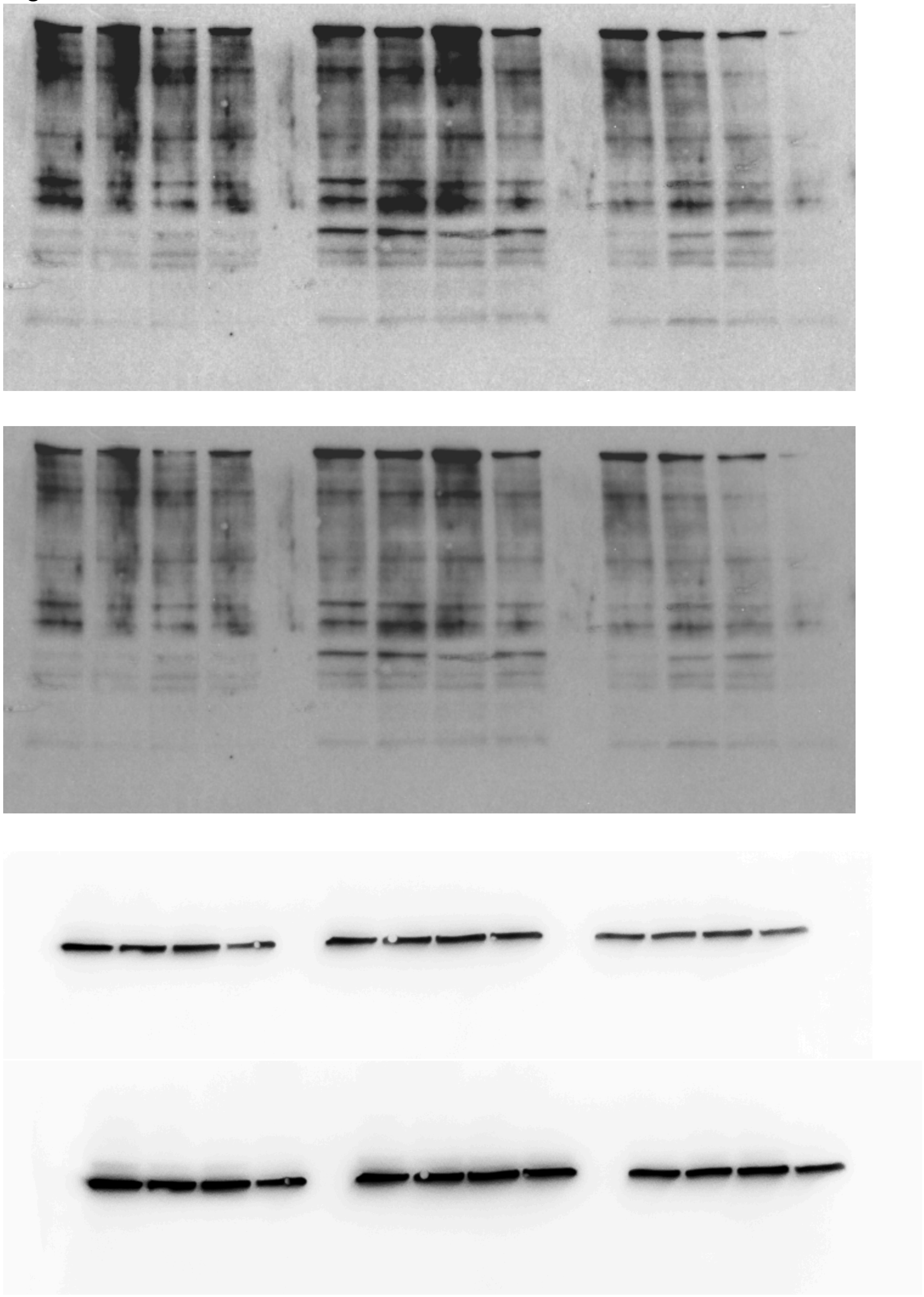

Figure 1L

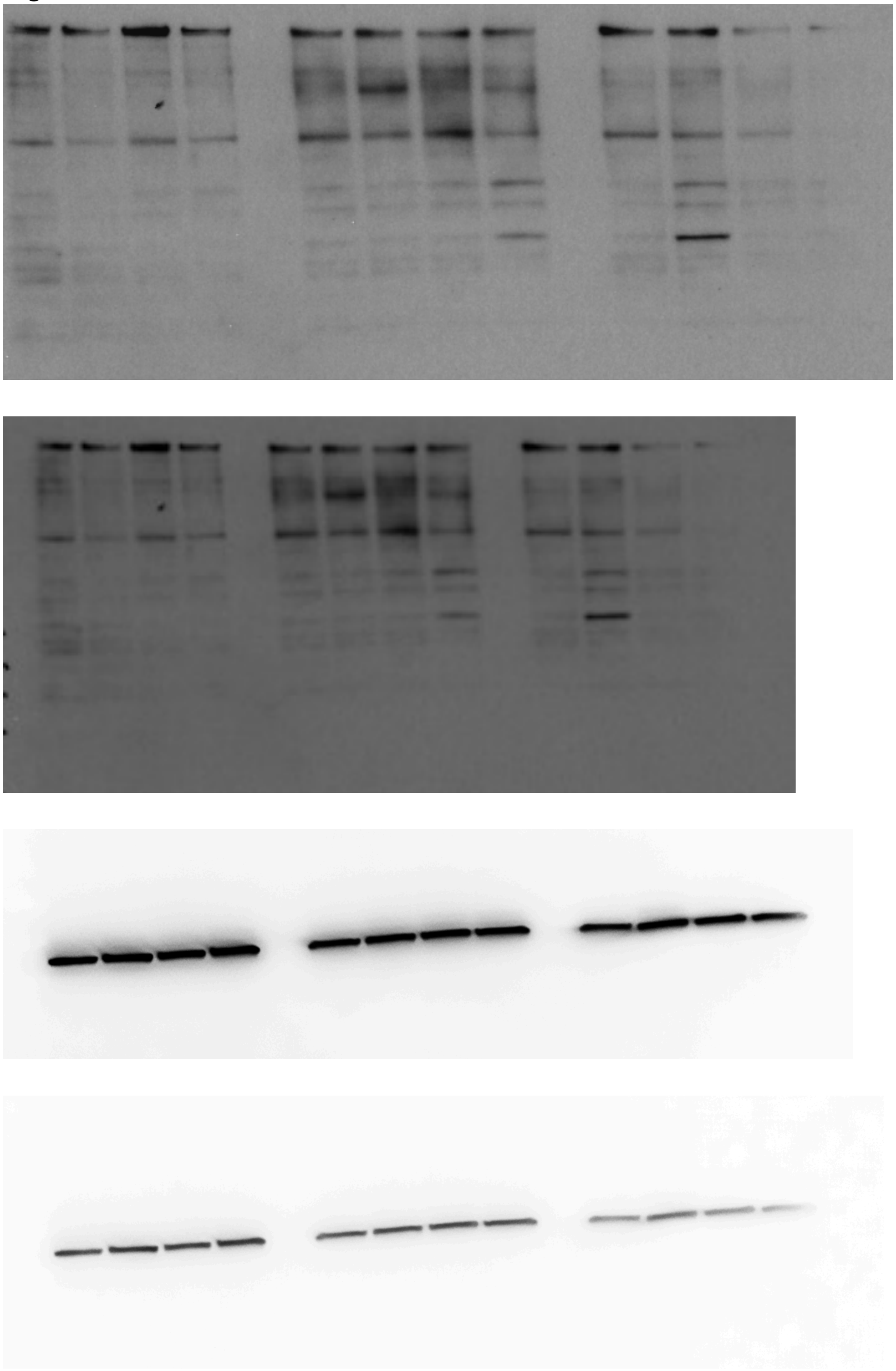

Figure 2A-CHOP

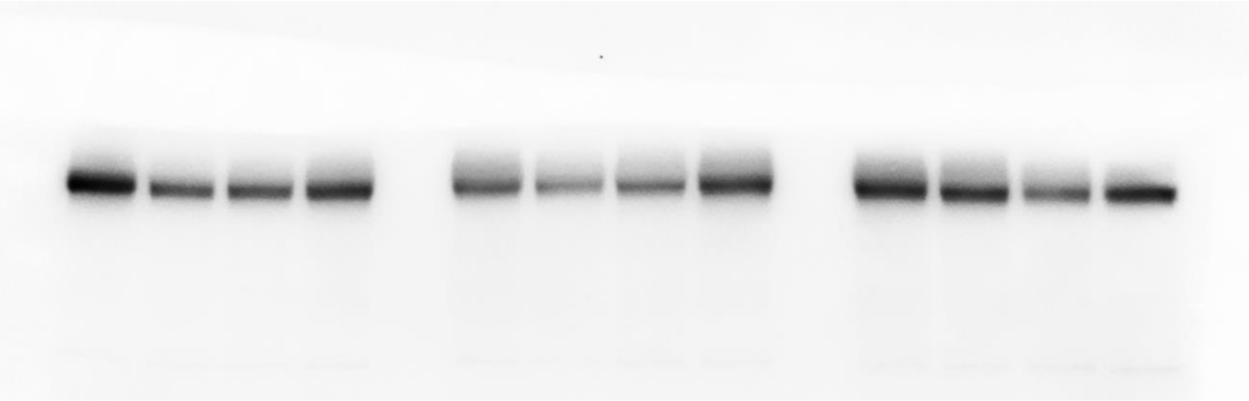

Exposure 7.5s

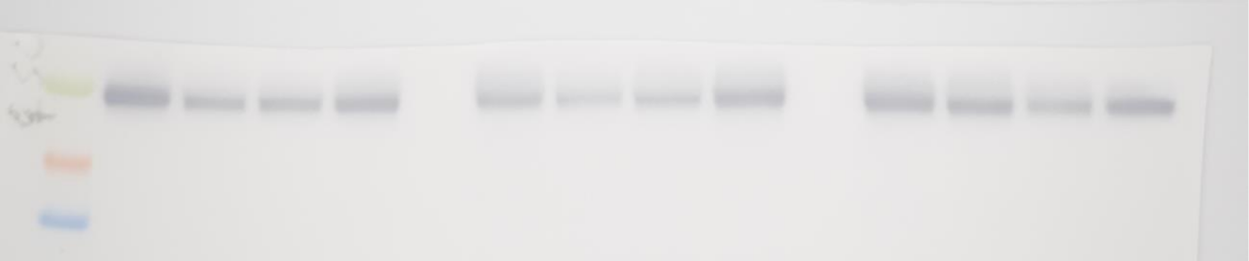

Exposure 7.5s

Figure 2A-ATF4

Exposure 3.7s

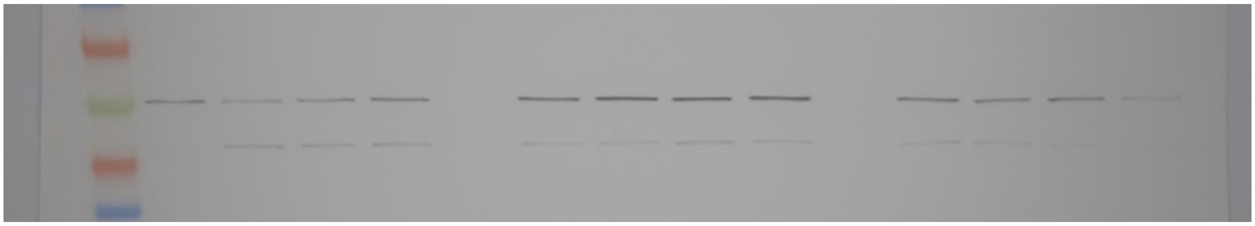

Exposure 7.5s

Figure 2A-ATF5

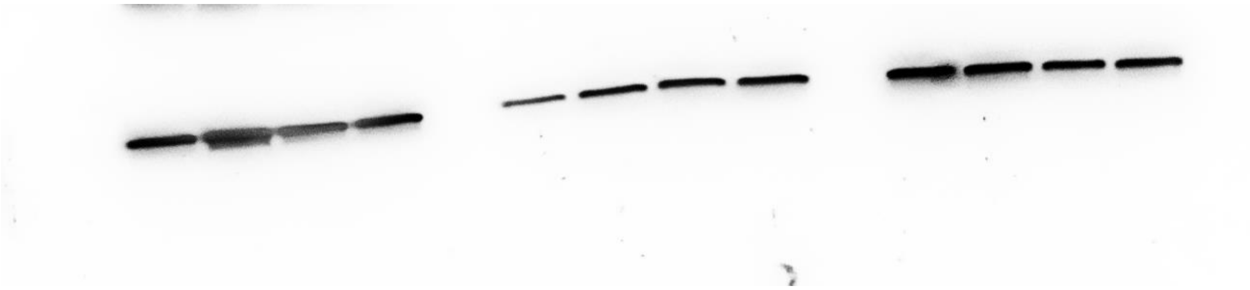

Exposure 3.5s

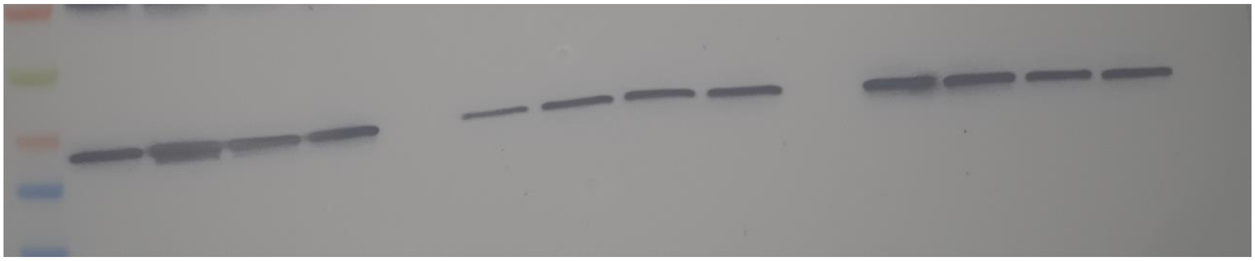

Exposure 3.5s

Figure 2A-LonP

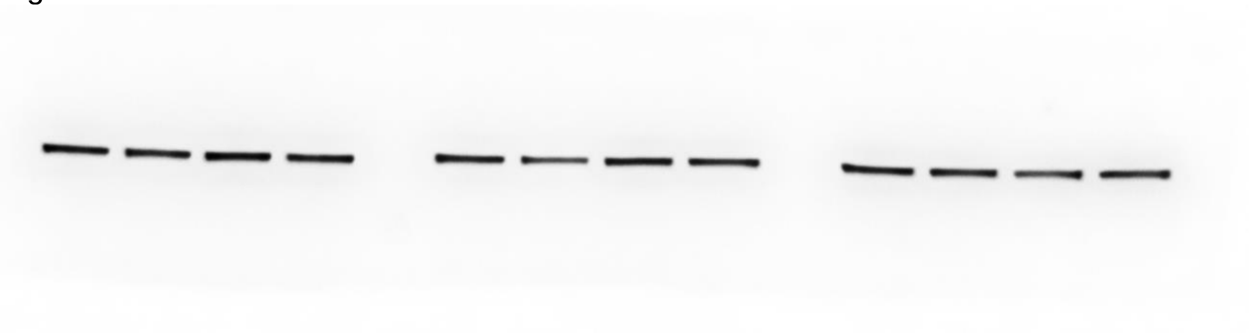

Exposure 3.7s

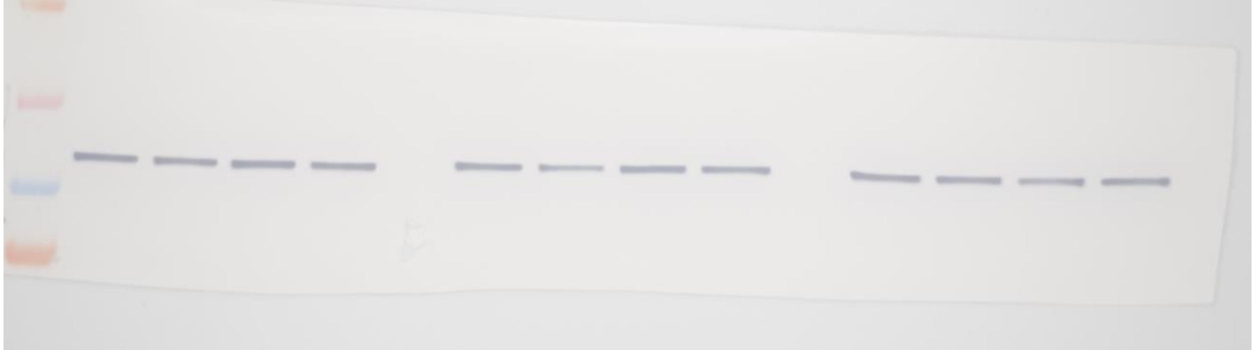

Exposure 3.7s

Figure 2A-actin

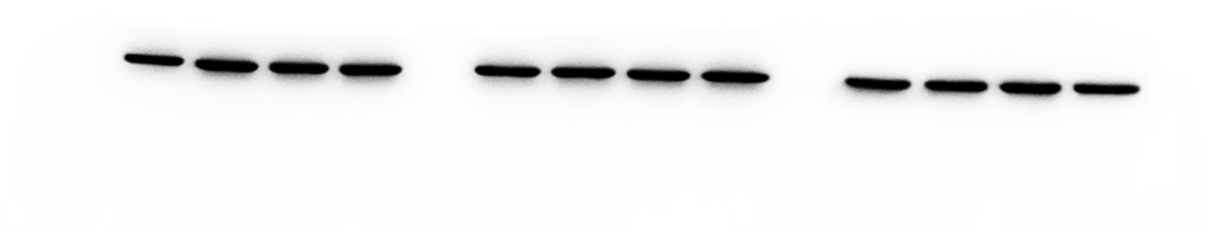

Exposure 3.7s

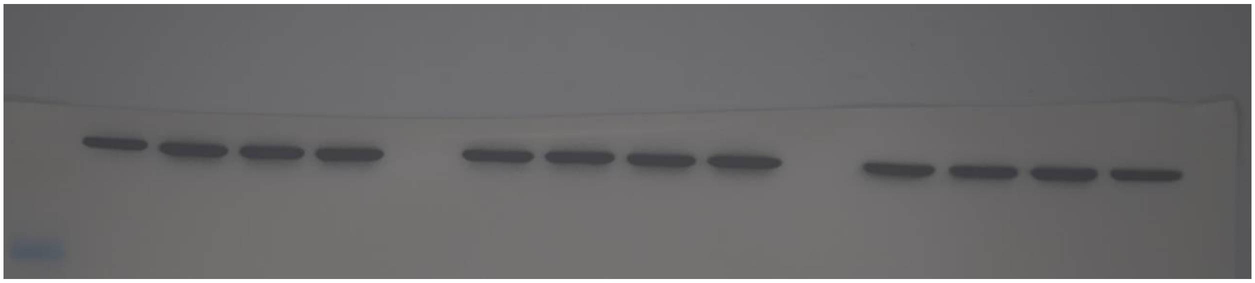

Exposure 3.7s

Figure 2BCHOP

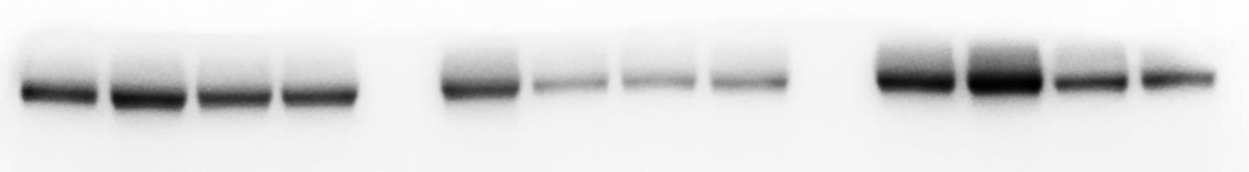

Exposure 7.5s

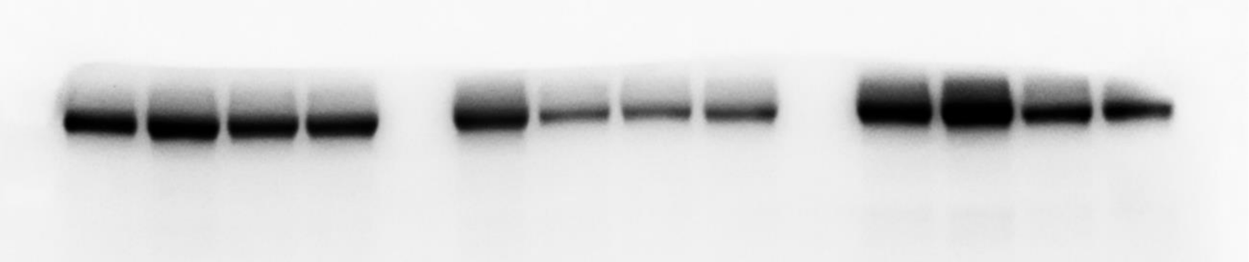

Exposure 14s

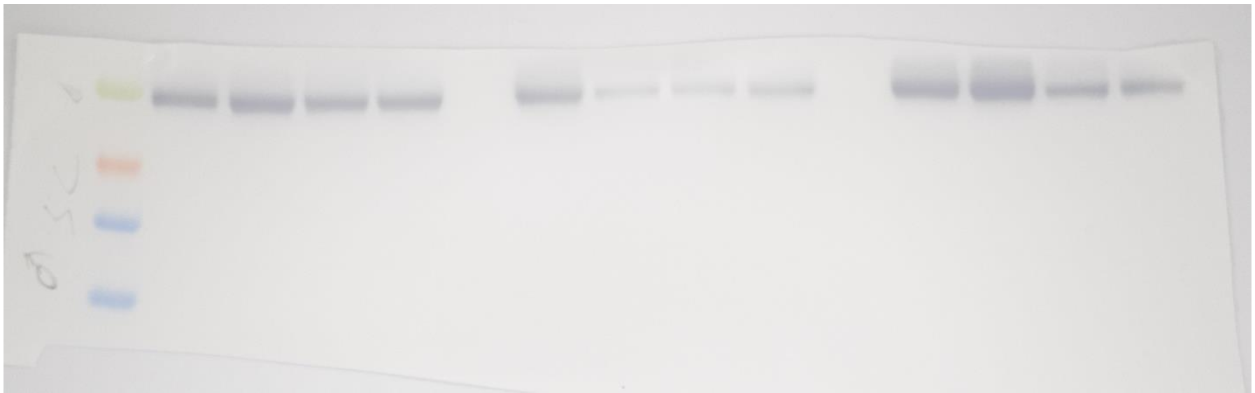

Exposure 14s

Figure 2BATF4

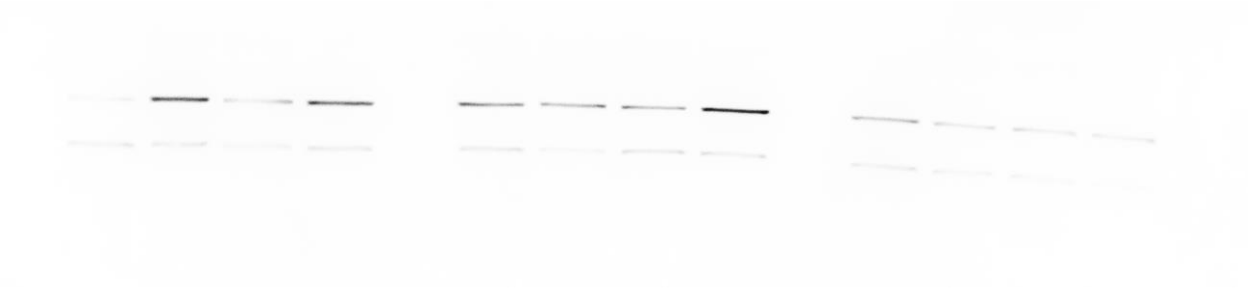

Exposure 3.7s

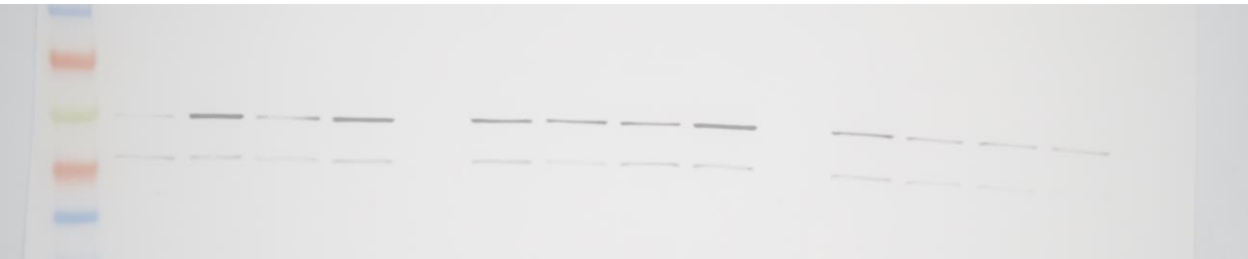

Exposure 7.5s

Figure 2BATF5

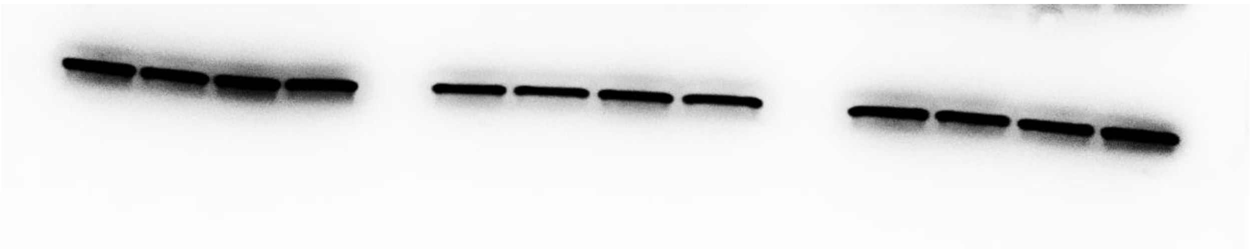

Exposure 1.8s

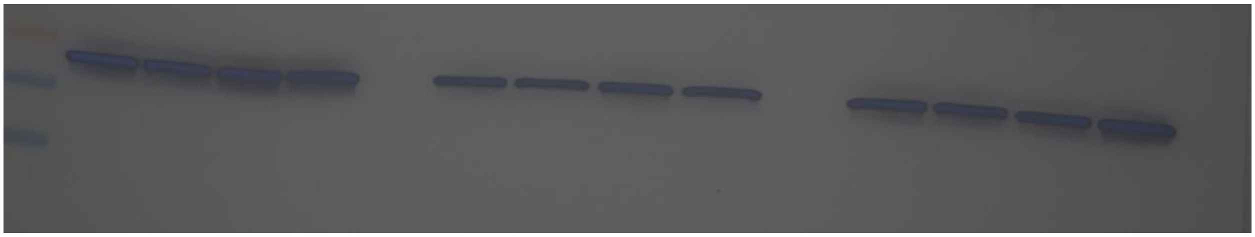

Exposure 1.8s

Figure 2BLonP

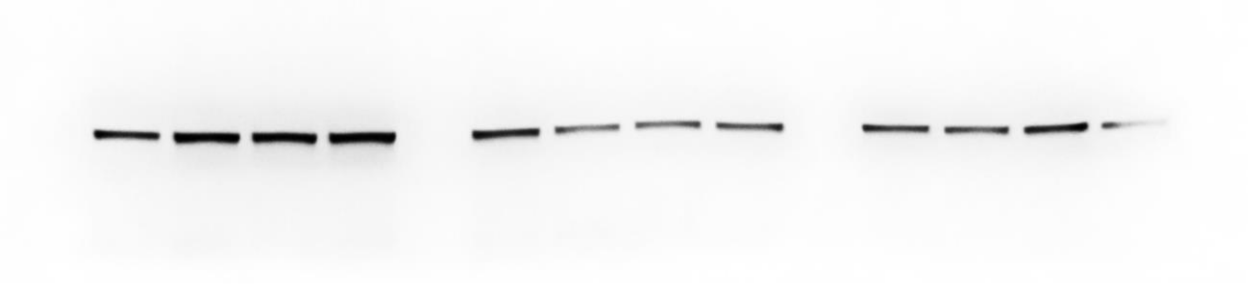

Exposure 1s

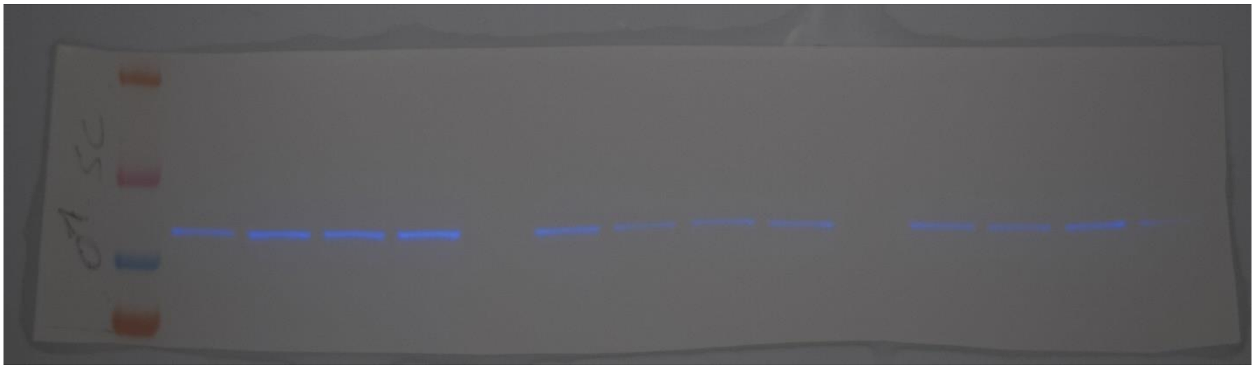

Exposure 1s

Figure 2B-actin

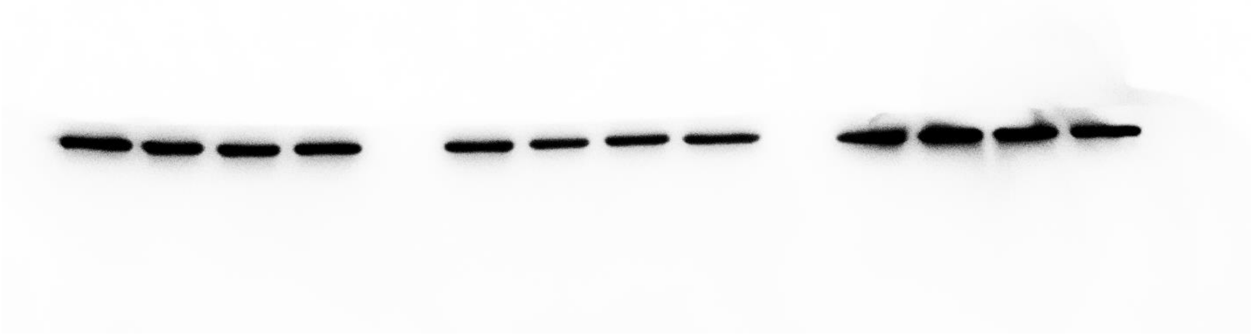

Exposure 3.7s

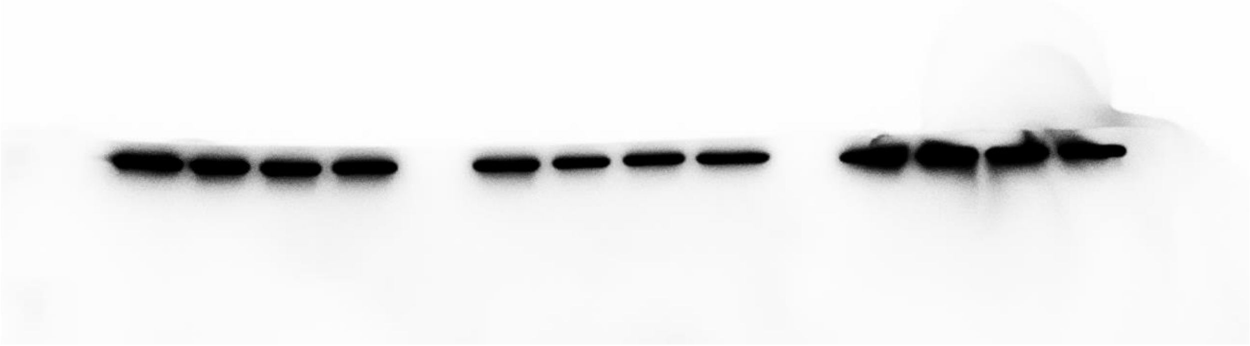

Exposure 7.5s

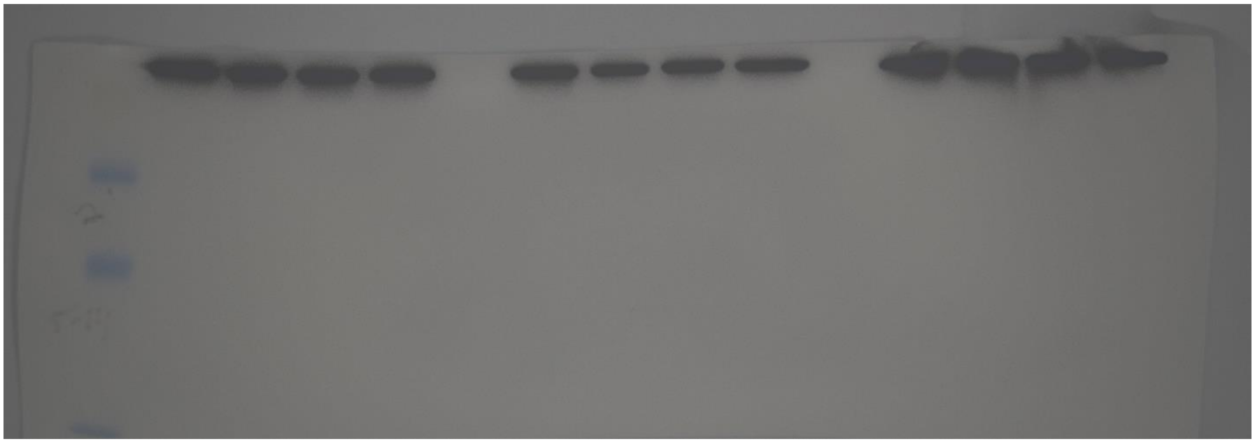

Exposure 7.5s

Figure 2D SIRT3

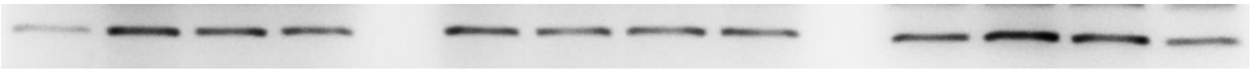

Exposure 27s

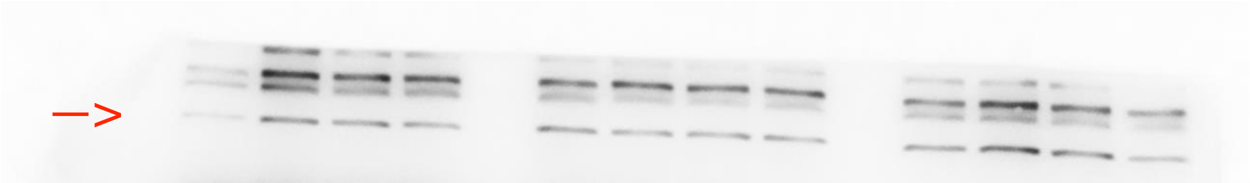

Exposure 14s

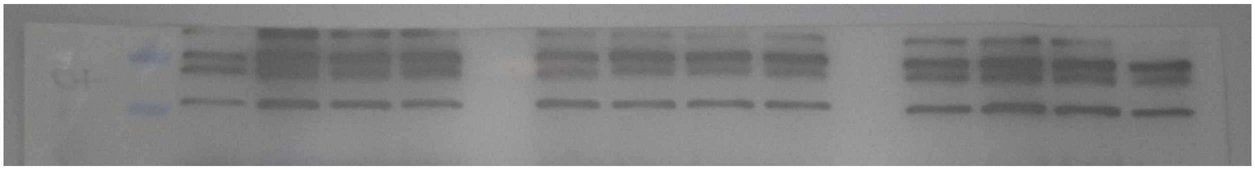

Exposure 27s

Figure 2DFoxo3a

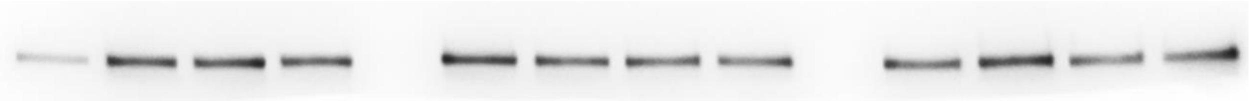

Exposure 7.5s

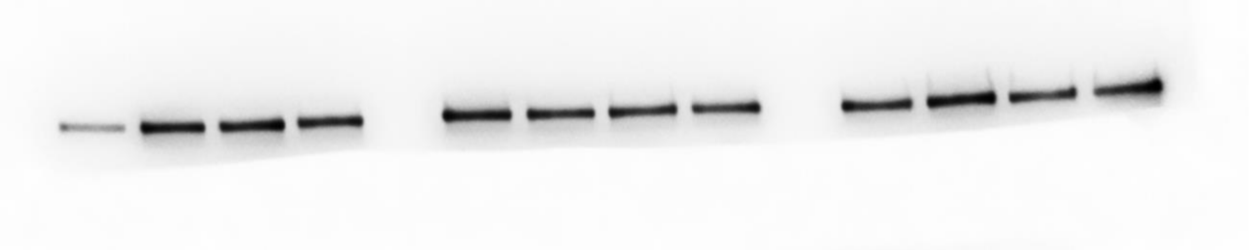

Exposure 14s

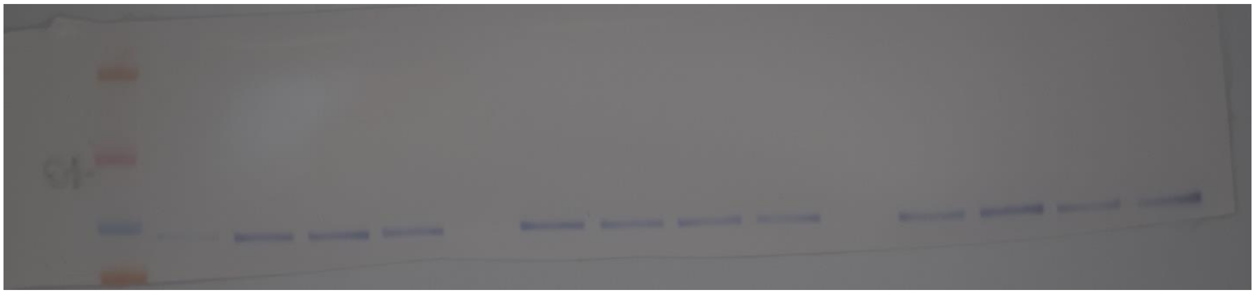

Exposure 7.5s

Figure 2D SOD2

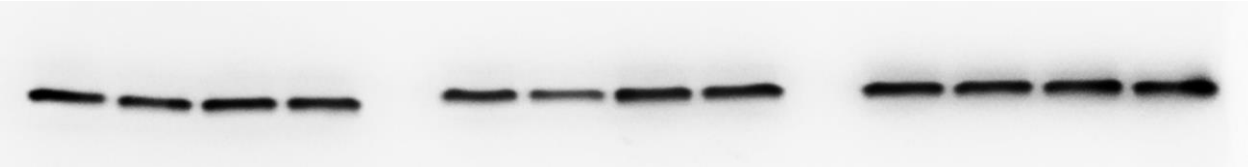

Exposure 3.7s

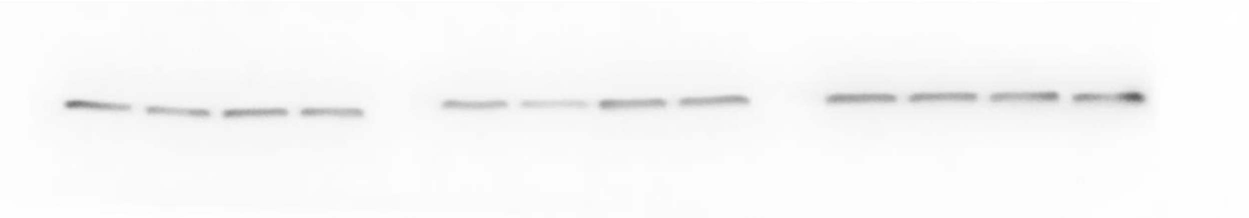

Exposure 1s

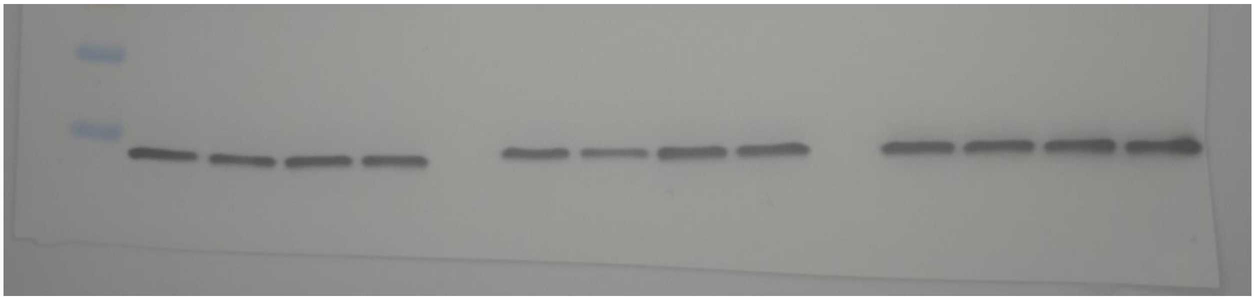

Exposure 3.7s

Figure 2DLC3b

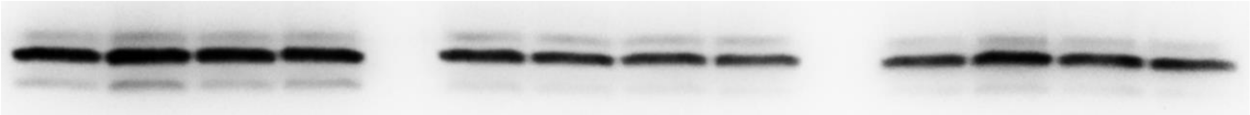

Exposure 14s

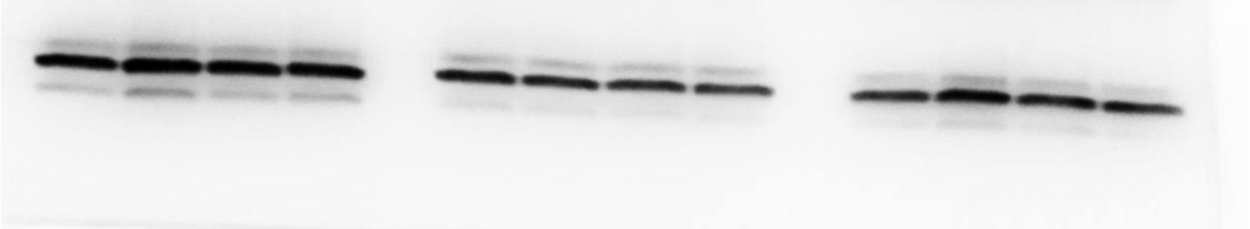

Exposure 14s

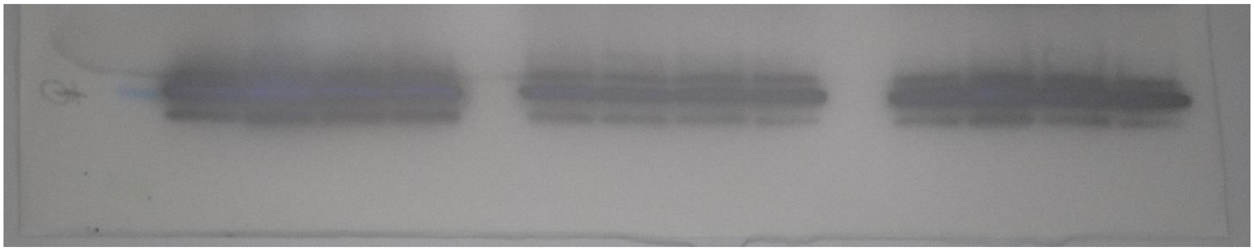

Exposure 14s

Figure 2D actin

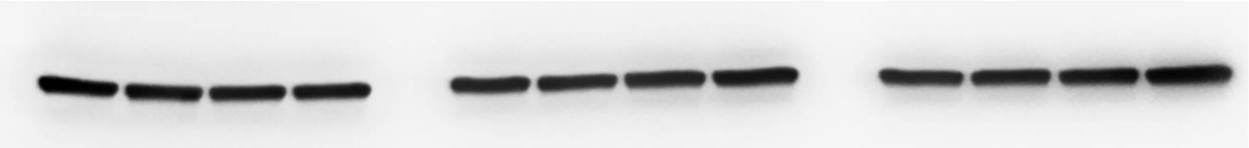

Exposure 7.5s

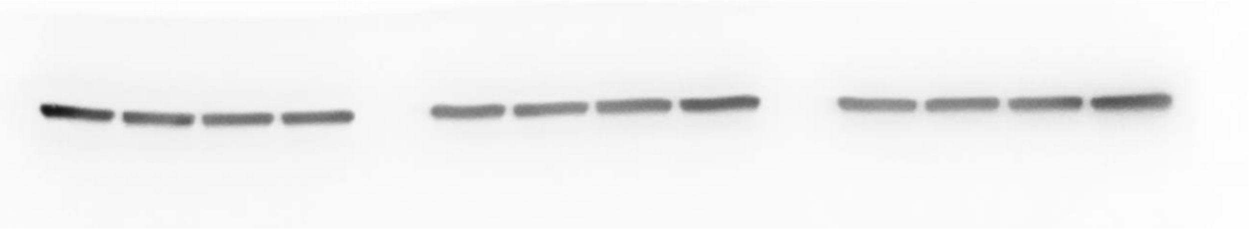

Exposure 3.7s

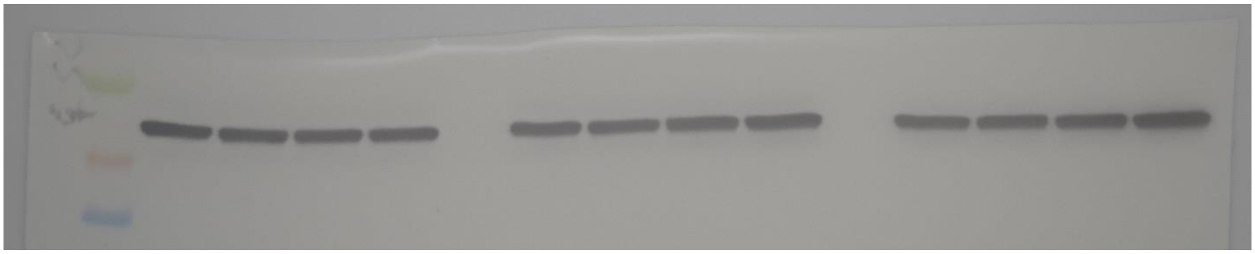

Exposure 7.5s

Figure 2E SIRT3

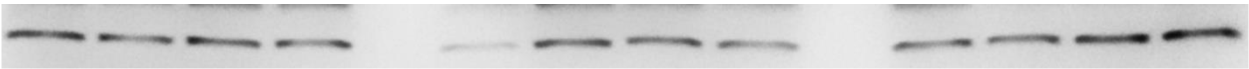

Exposure 56s

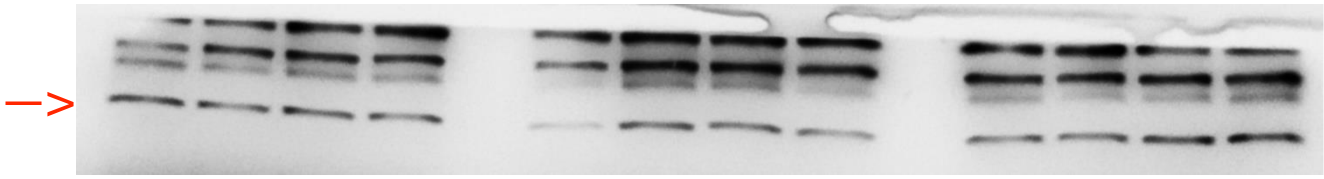

Exposure 56s

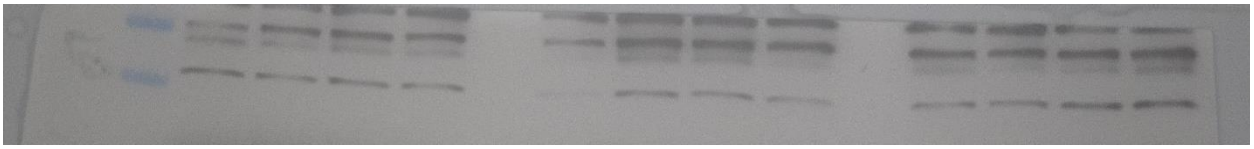

Exposure 56s

Figure 2E Foxo3a

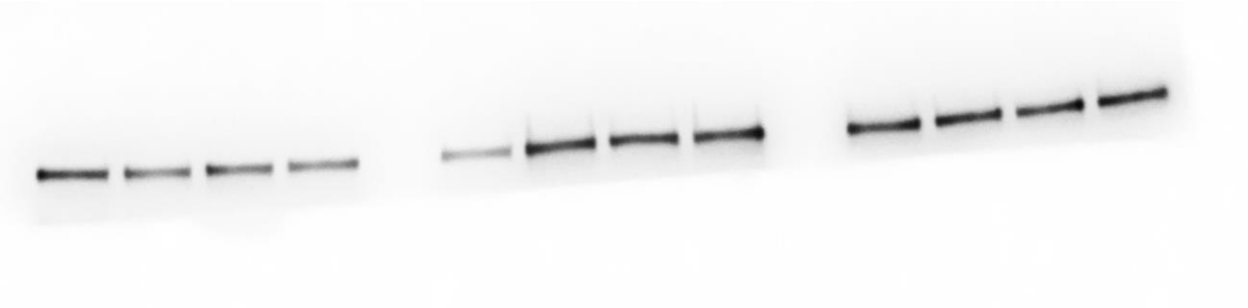

Exposure 6.5s

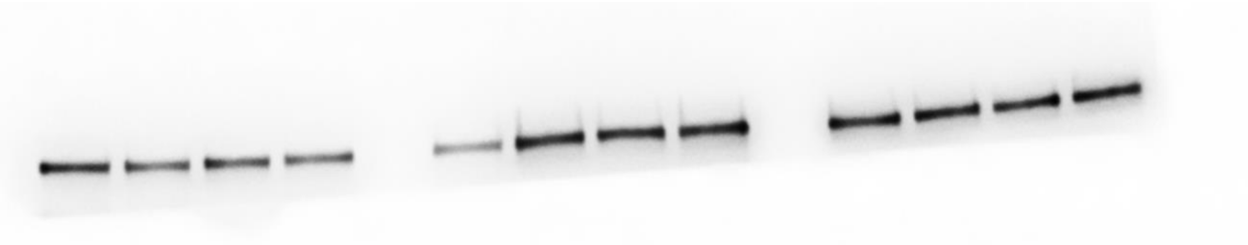

Exposure 7.5s

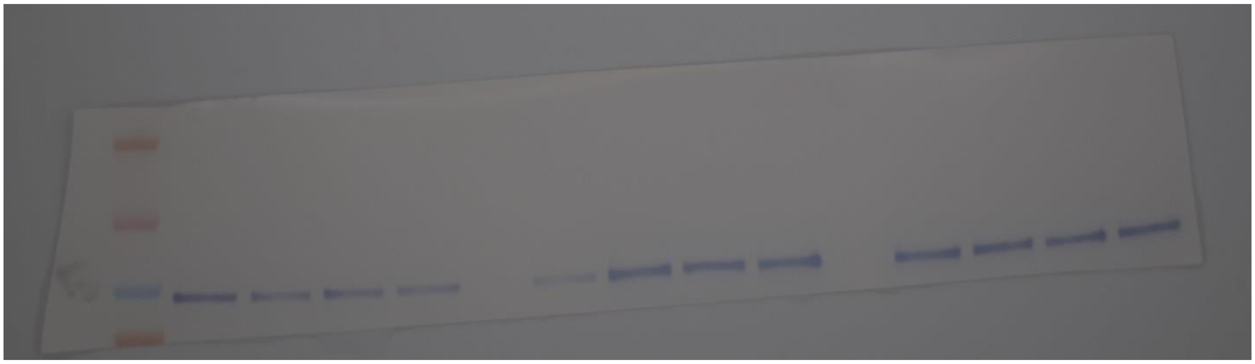

Exposure 7.5s

Figure 2E SOD2

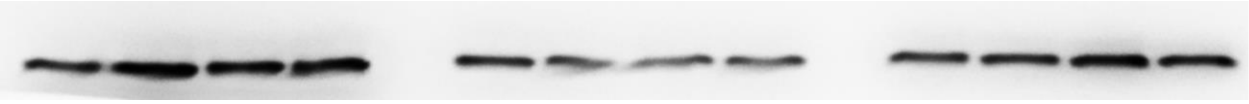

Exposure 3.7s

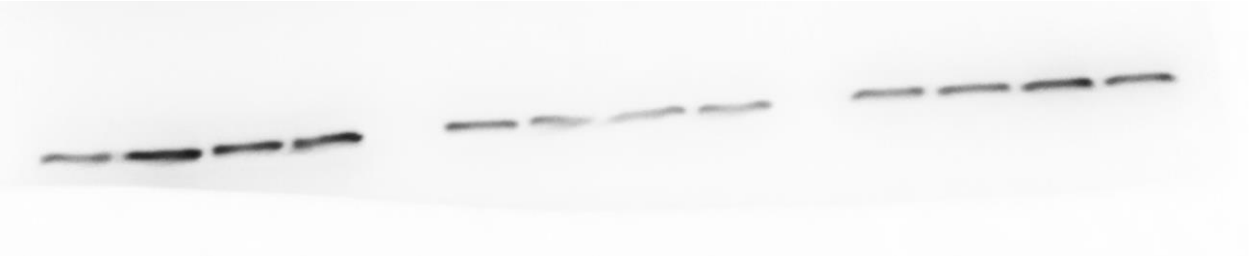

Exposure 1.8s

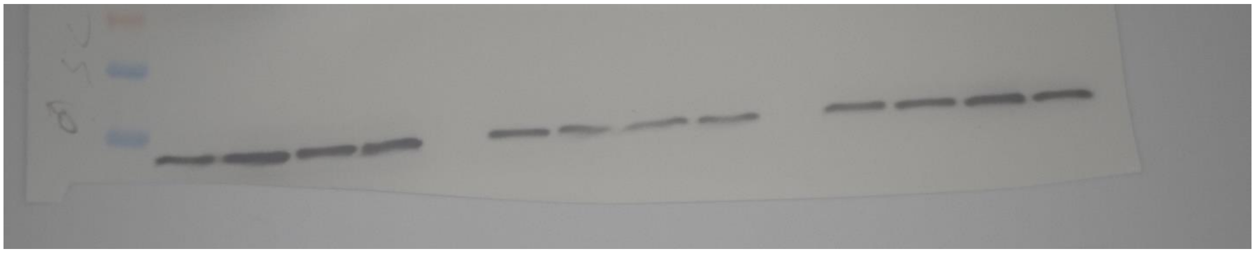

Exposure 3.7s

Figure 2E LC3b

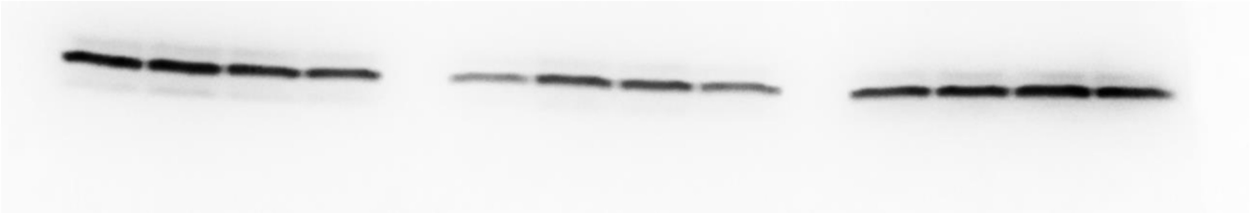

Exposure 14s

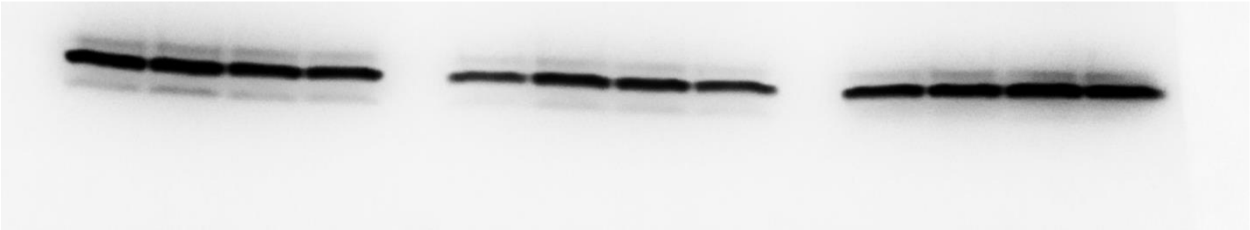

Exposure 27s

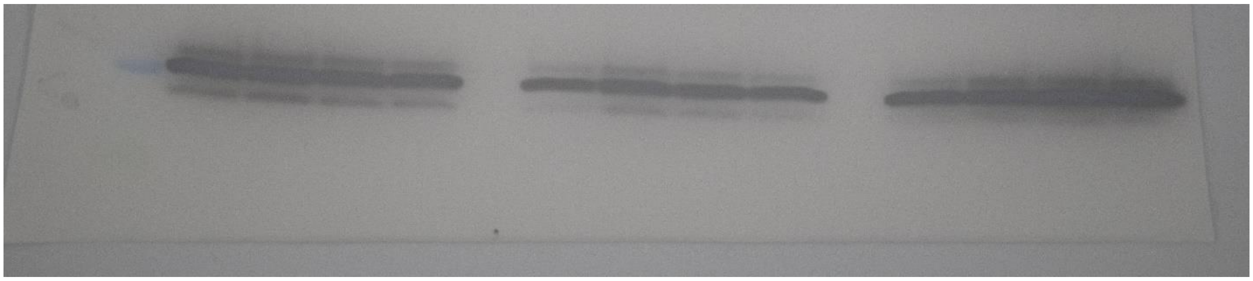

Exposure 27s

Figure 2E actin

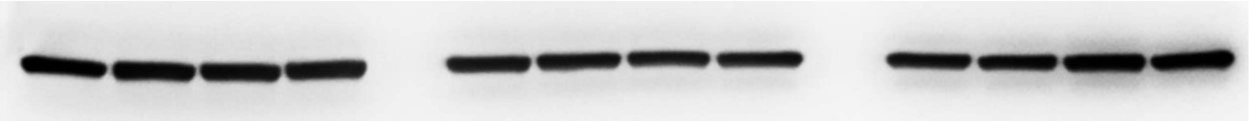

Exposure 7.5s

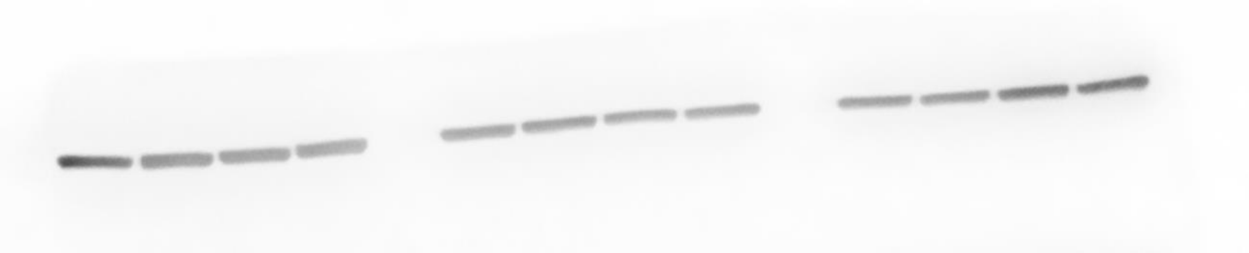

Exposure 3.7s

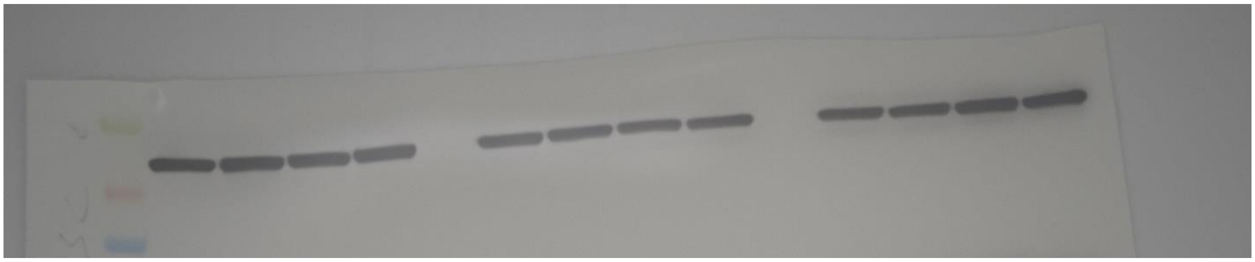

Exposure 7.5s

Figure 2G-pERα

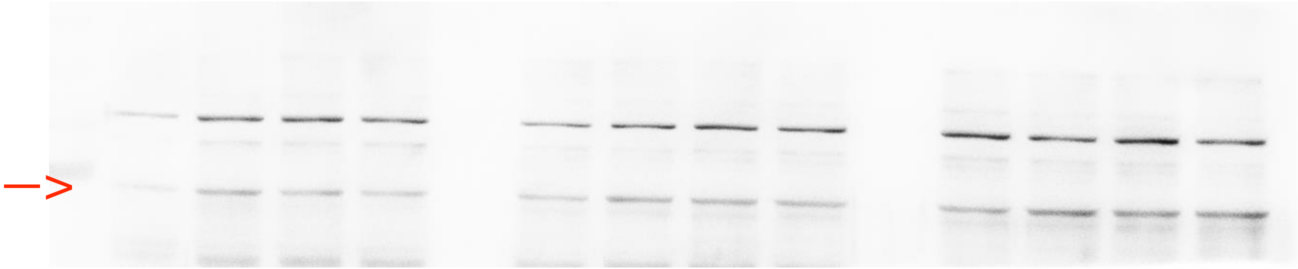

Exposure 1.8s

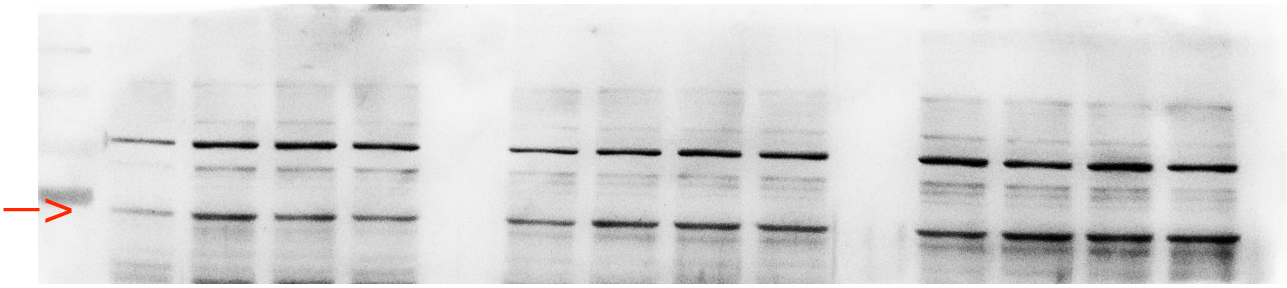

Exposure 3.7s

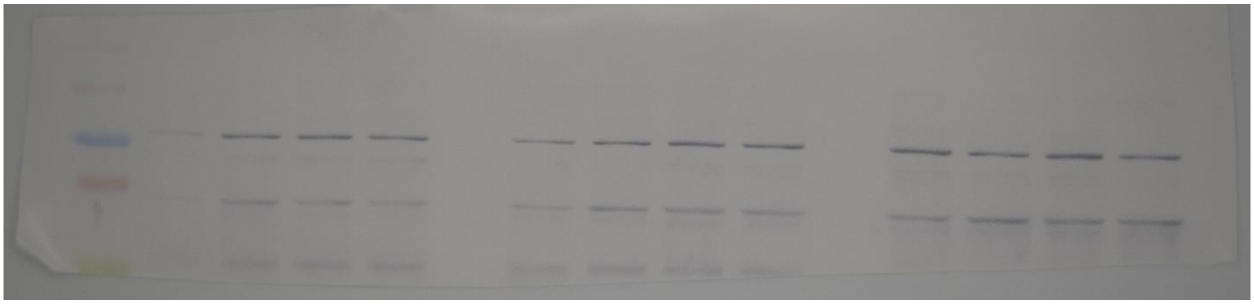

Exposure 1.8s

Figure 2G total ER α

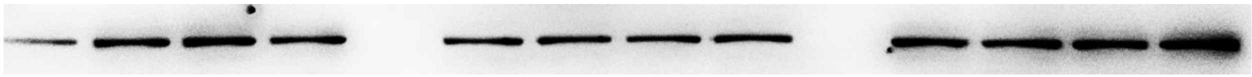

Exposure 27s

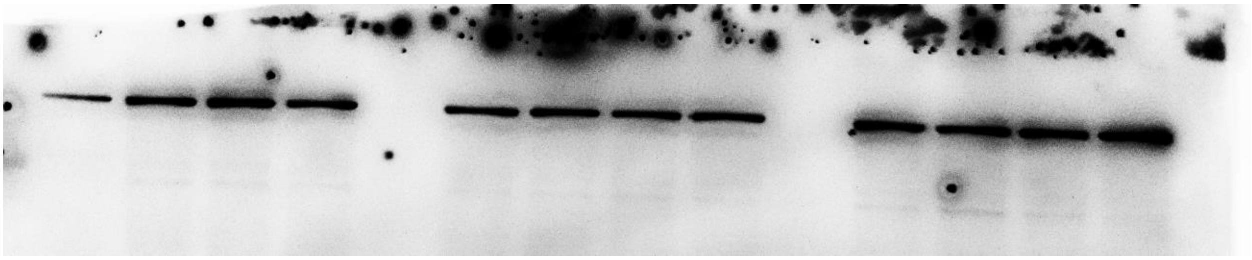

Exposure 56s

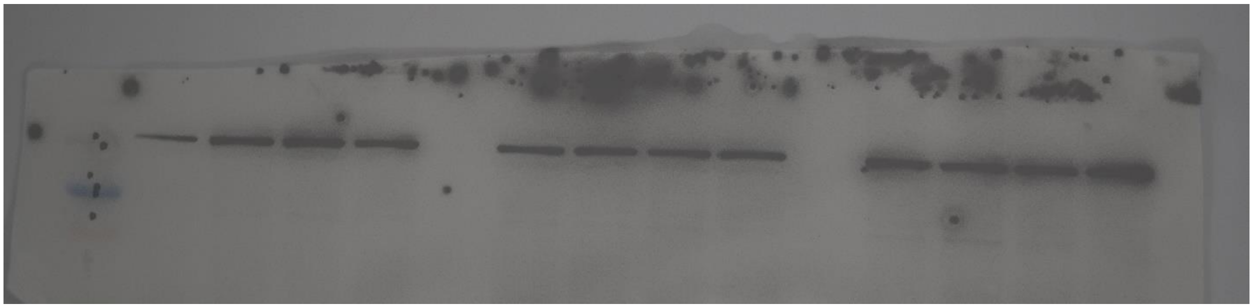

Exposure 56s

Figure 2G pAKT

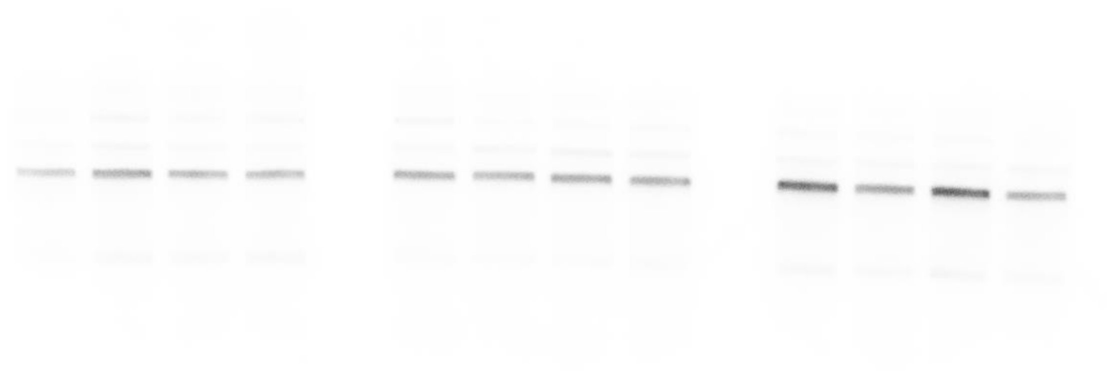

Exposure 3.7s

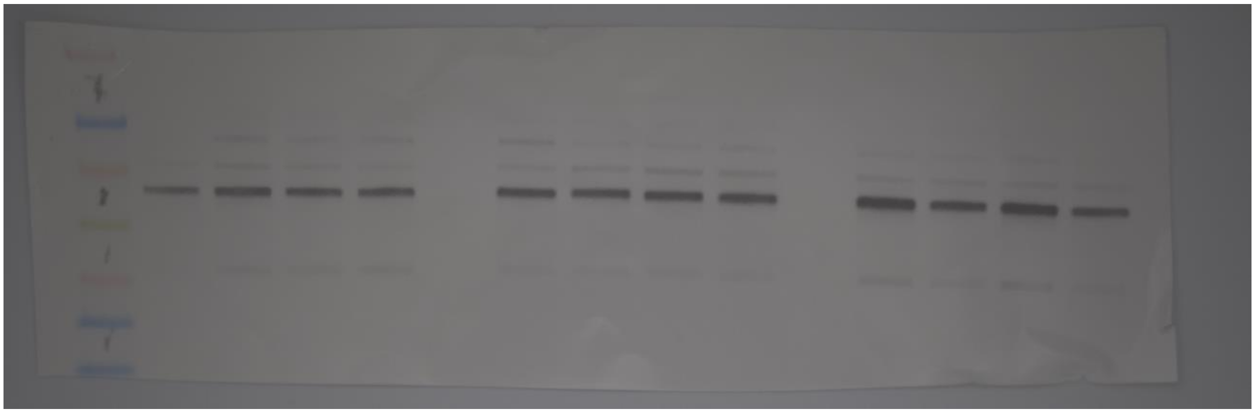

Exposure 14s

Figure 2G total AKT

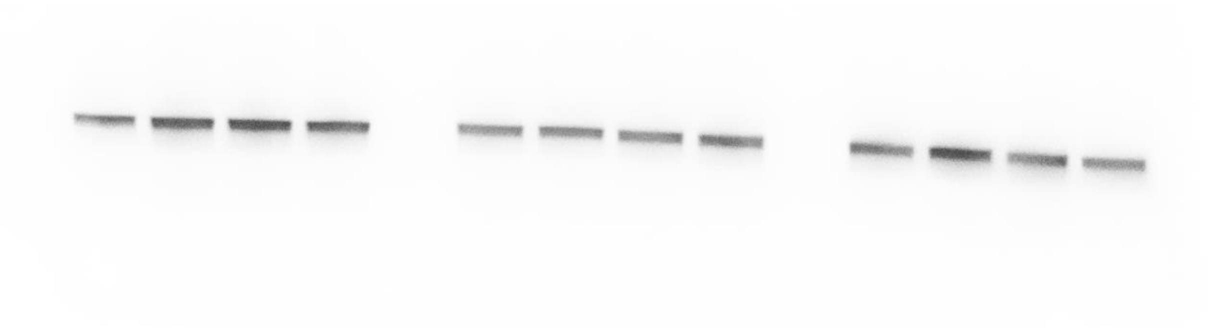

Exposure 3.7s

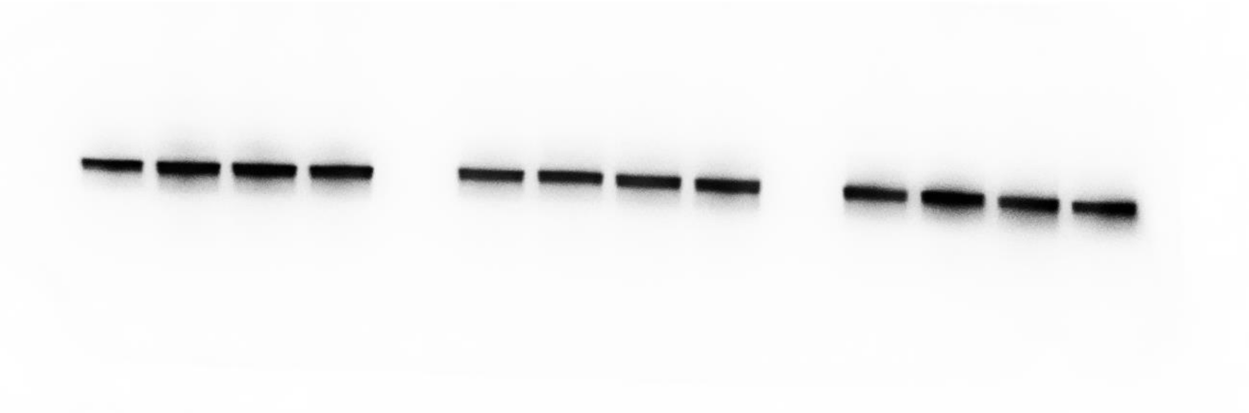

Exposure 7.5s

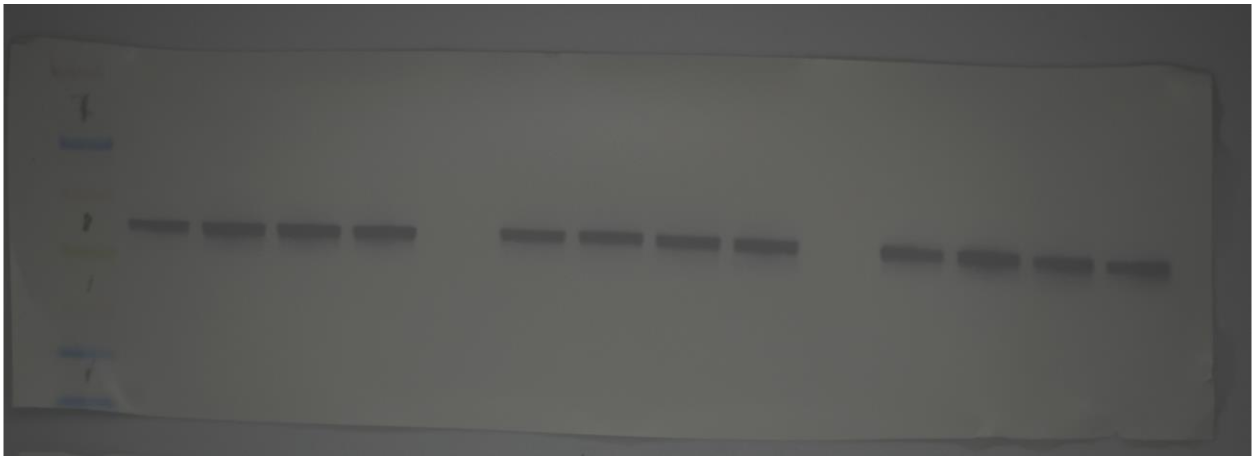

Exposure 7.5s

Figure 2G NRF1

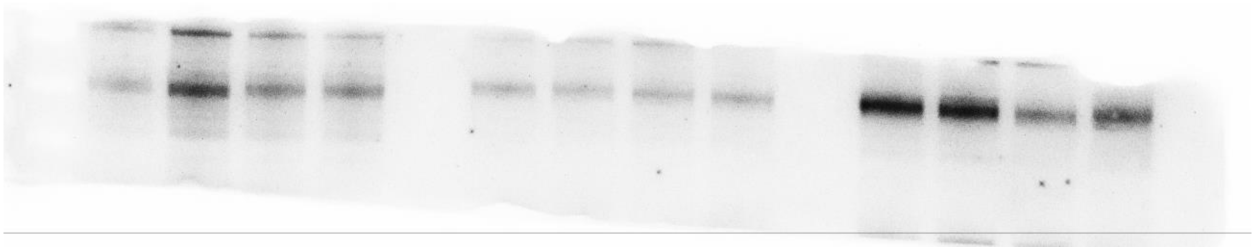

Exposure 120s

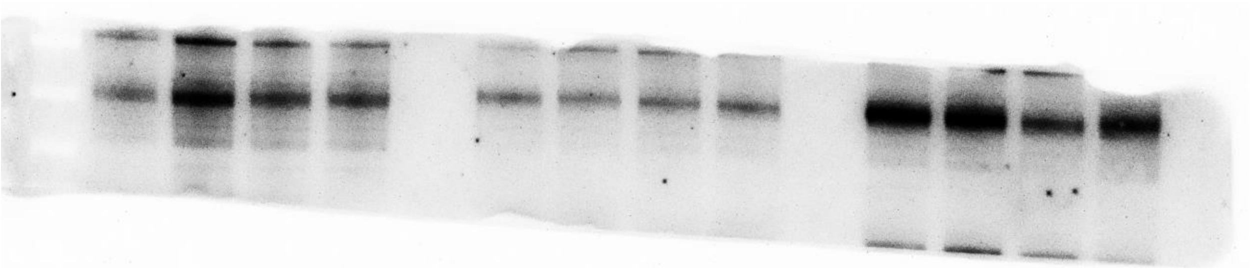

Exposure 240s

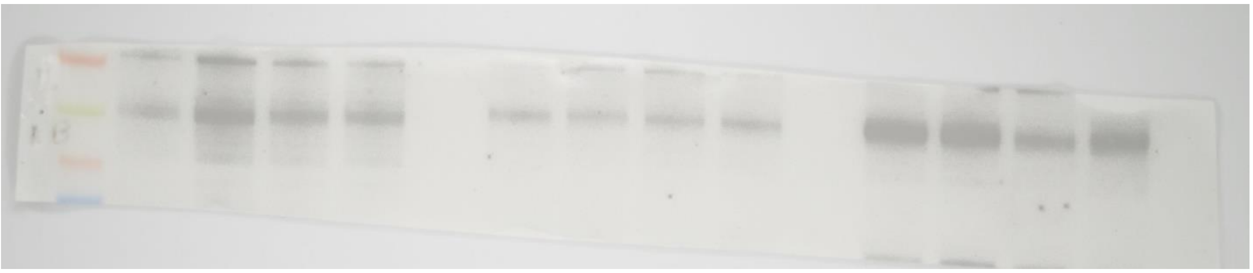

Exposure 240s

Figure 2G Omi

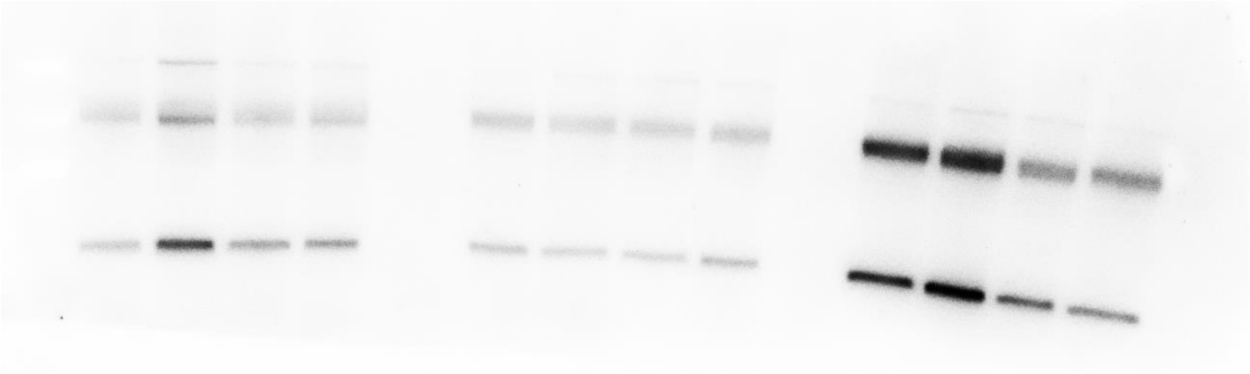

Exposure 56s

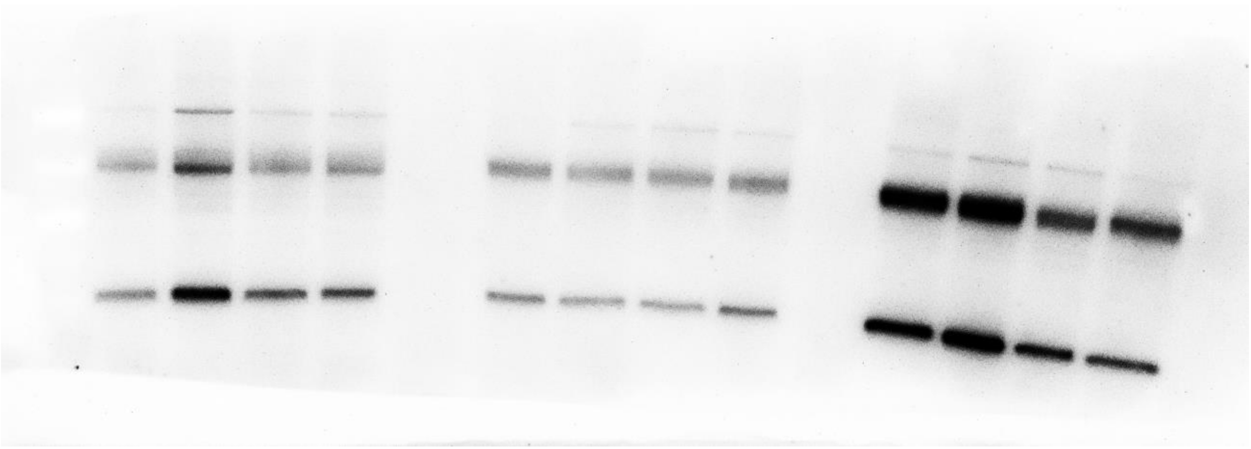

Exposure 120s

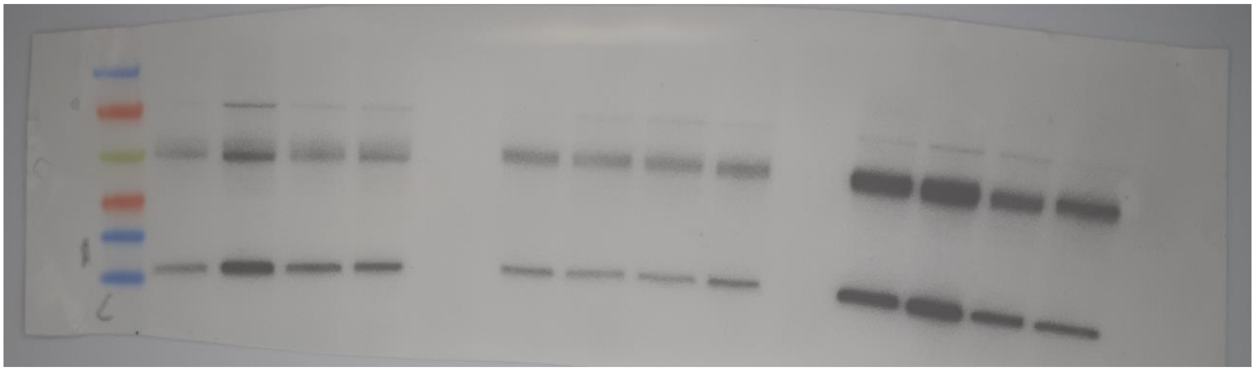

Exposure 120s

Figure 2H pERα

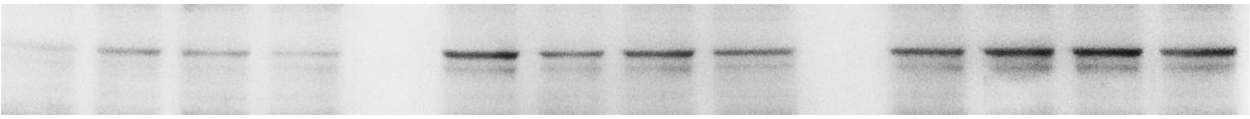

Exposure 1.8s

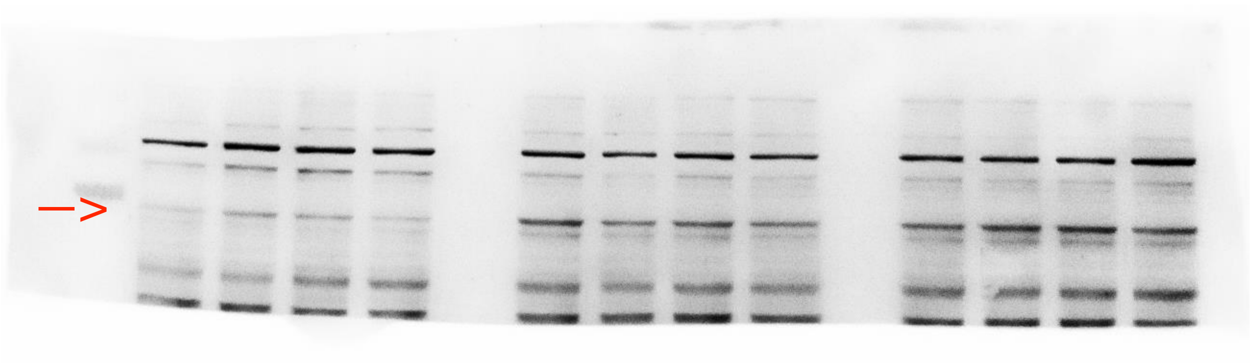

Exposure 1.8s

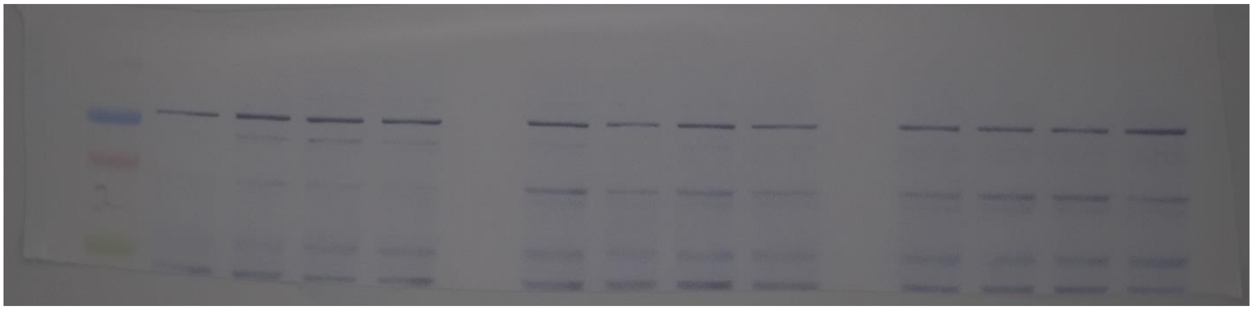

Exposure 1.8s

Figure 2H total ERα

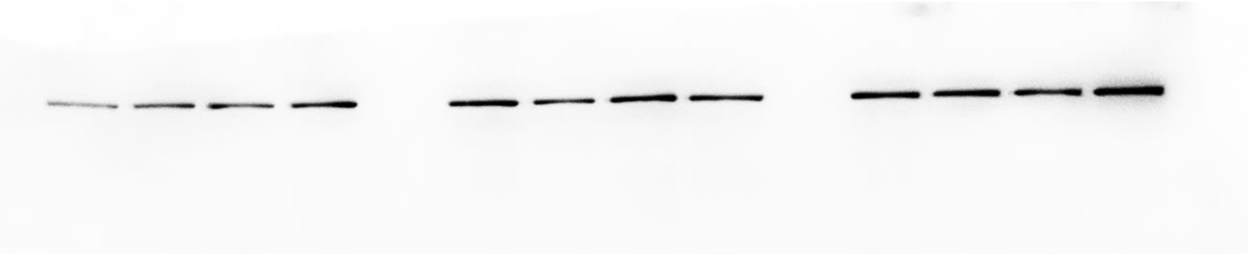

Exposure 14s

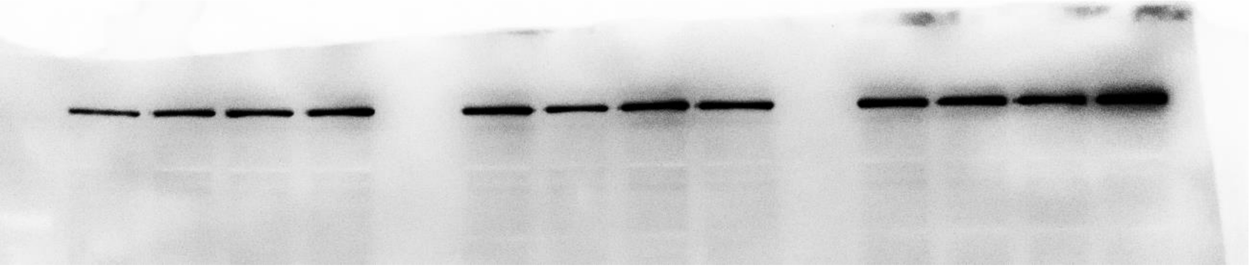

Exposure 27s

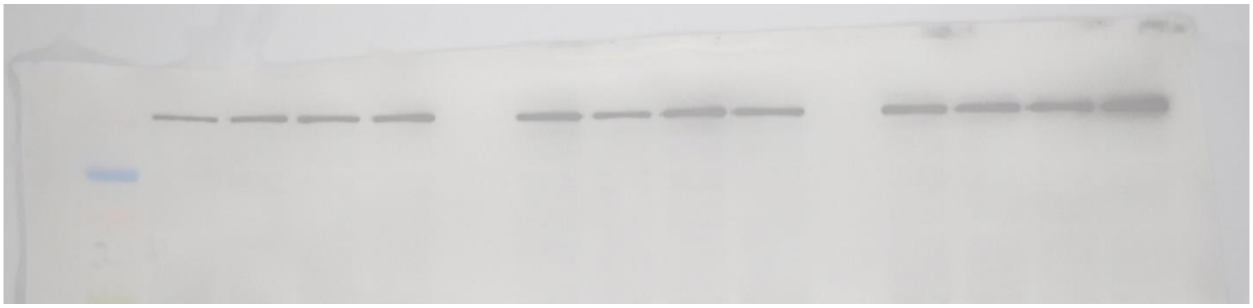

Exposure 27s

Figure 2H pAKT

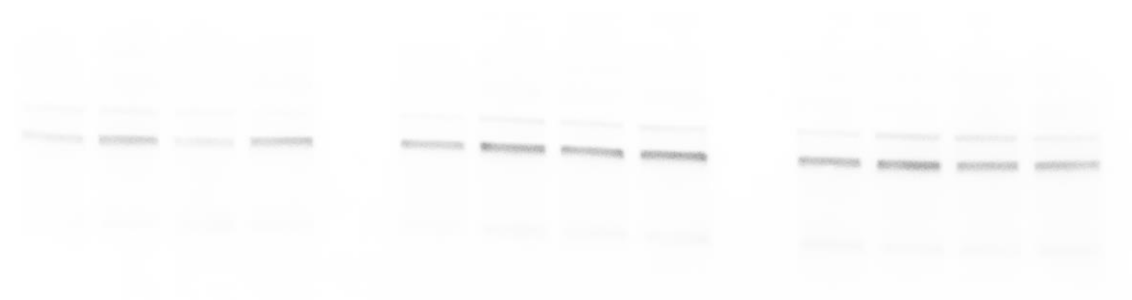

Exposure 7.5s

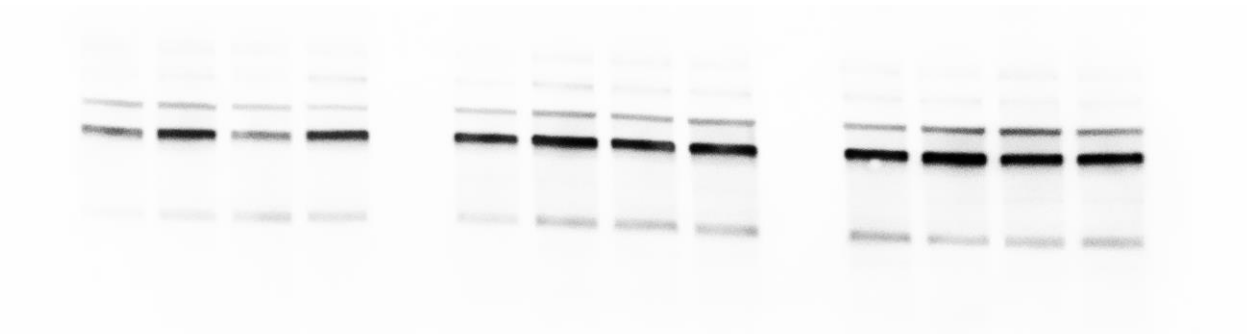

Exposure 27s

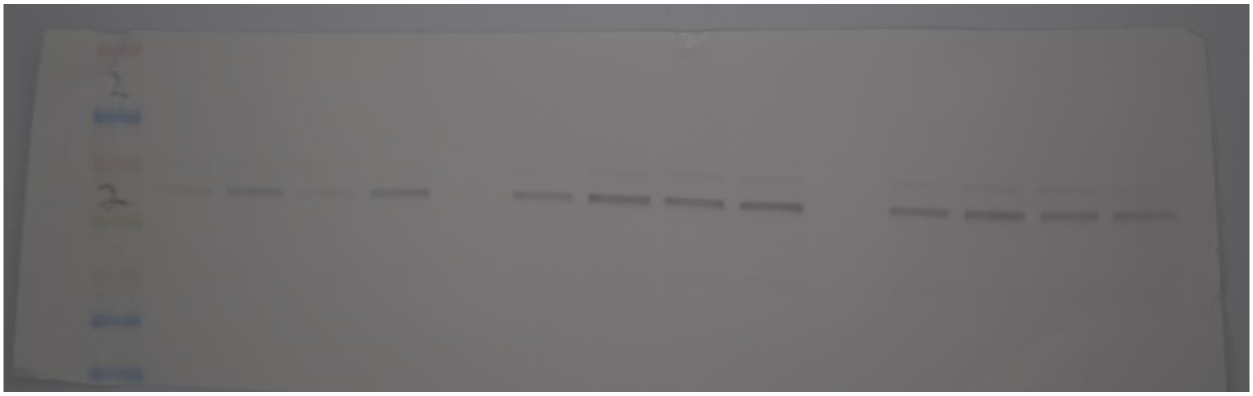

Exposure 27s

Figure 2H total AKT

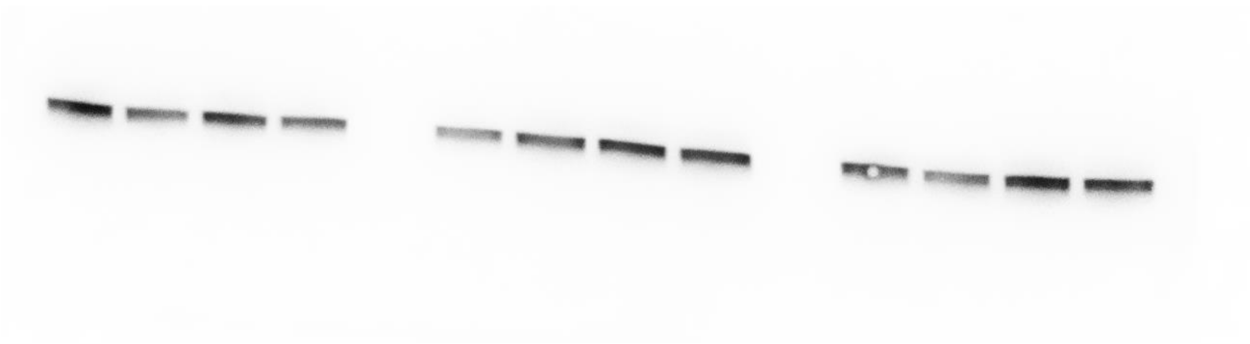

Exposure 7.5s

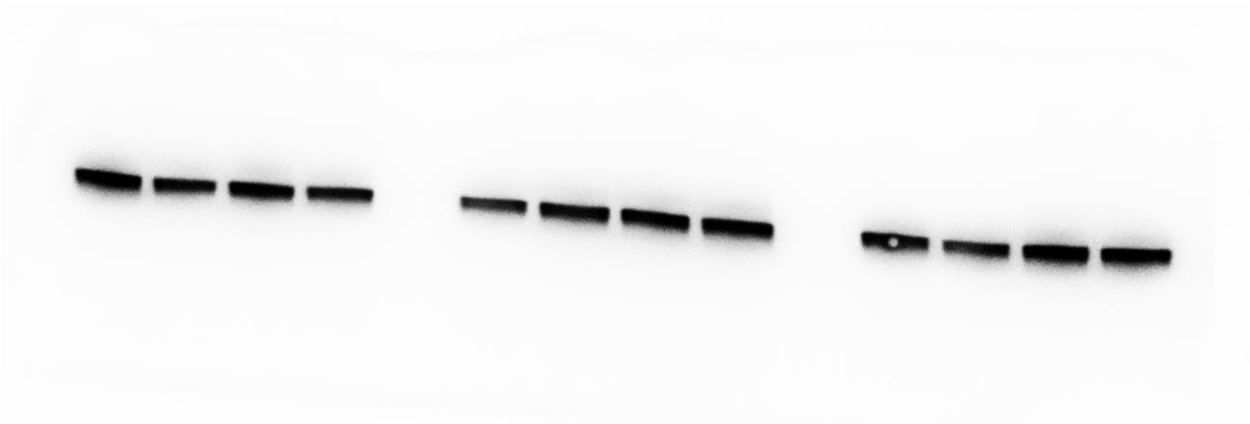

Exposure 14s

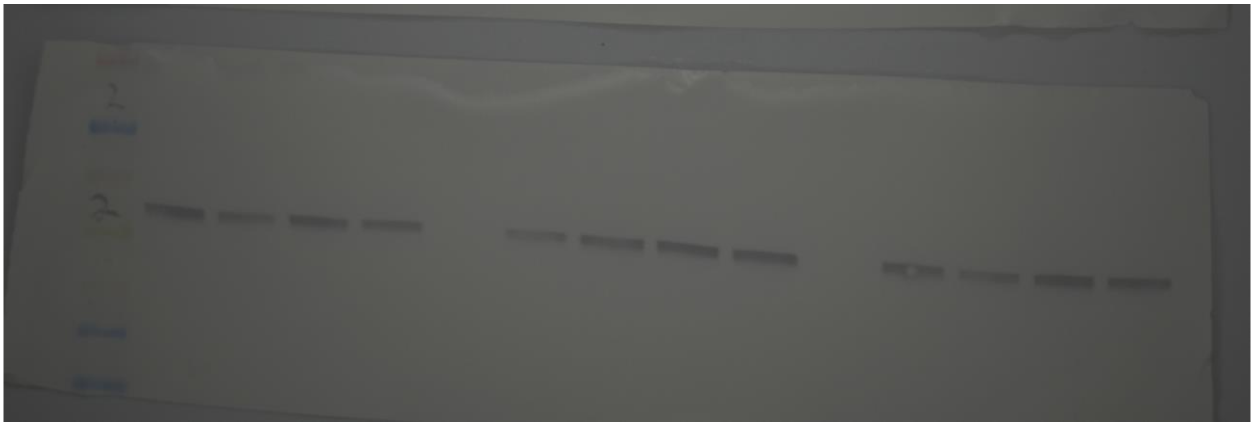

Exposure 14s

Figure 2H NRF1

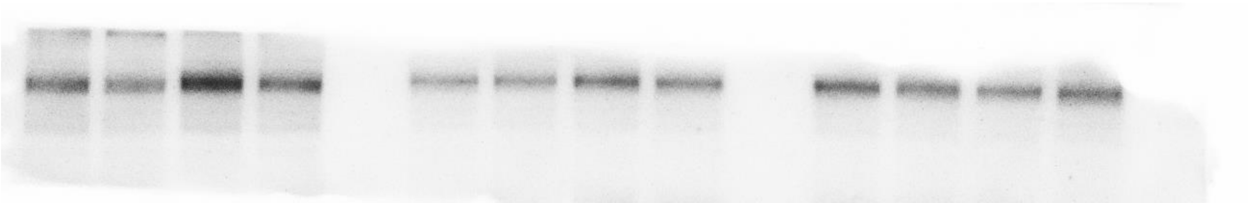

Exposure 120s

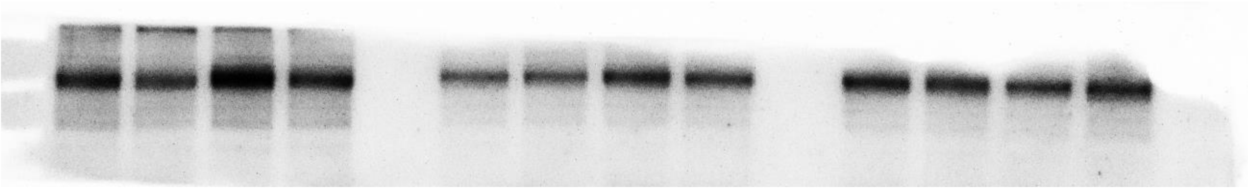

Exposure 240s

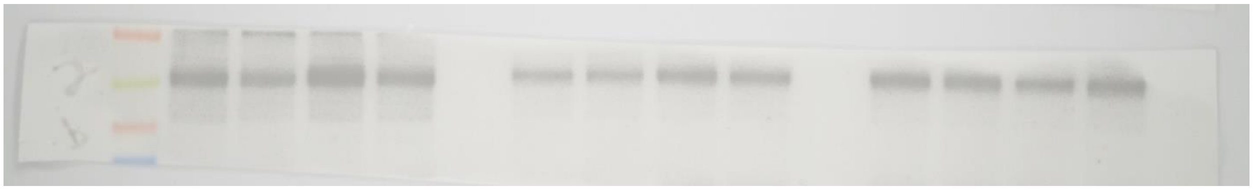

Exposure 240s

Figure 2H Omi

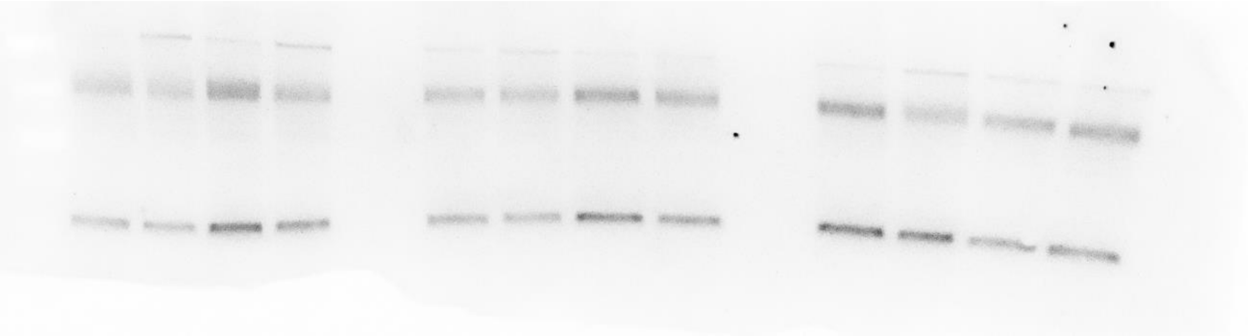

Exposure 120s

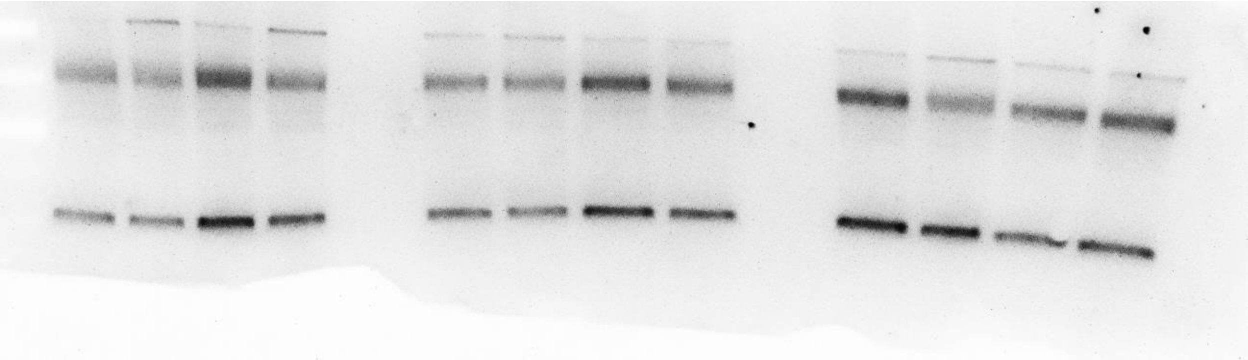

Exposure 240s

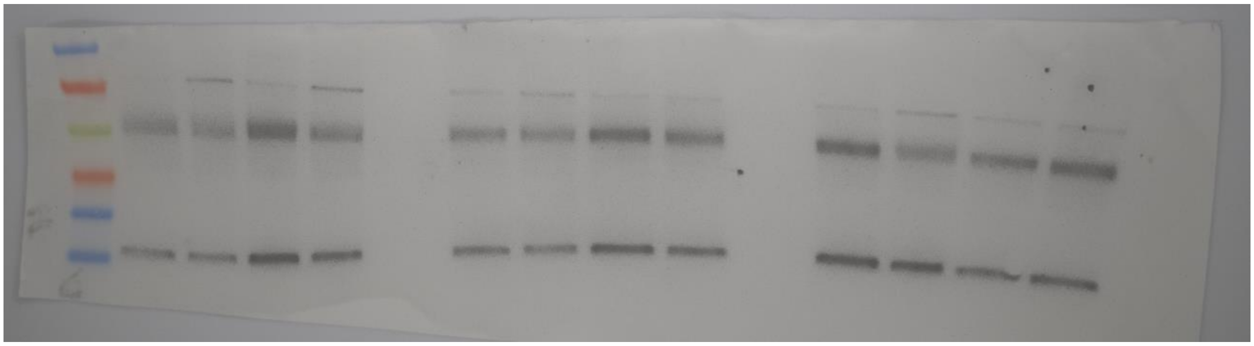

Exposure 240s

Figure 5C Ub K48

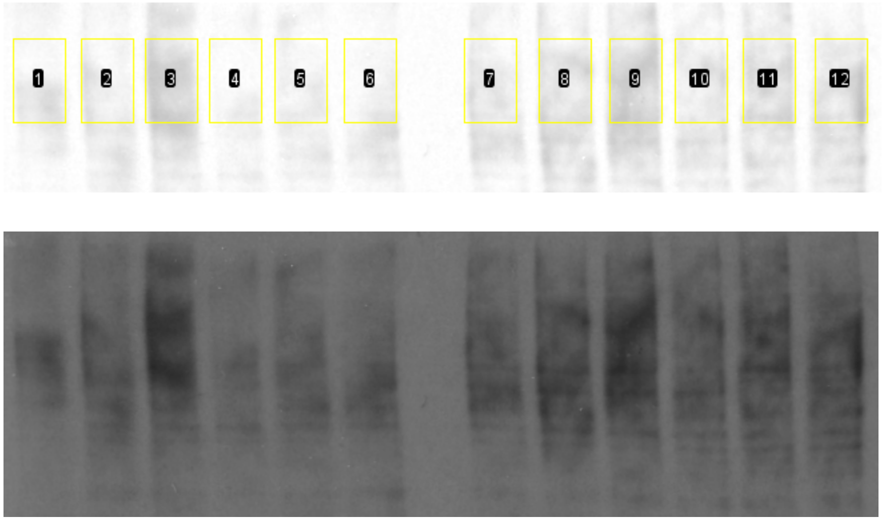

Figure 5C actin

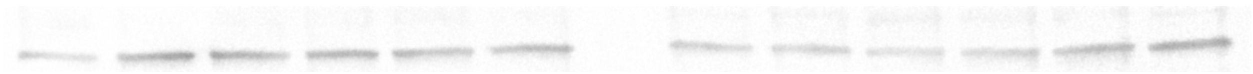

Exposure 3.5s

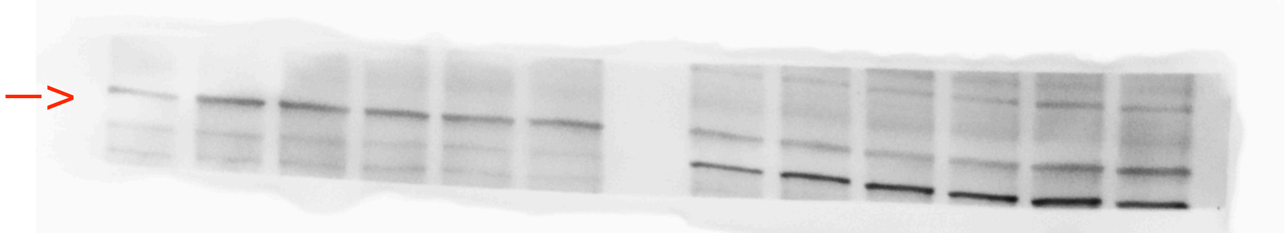

Exposure 7.5s

Supplementary figure 1 A Ub K48

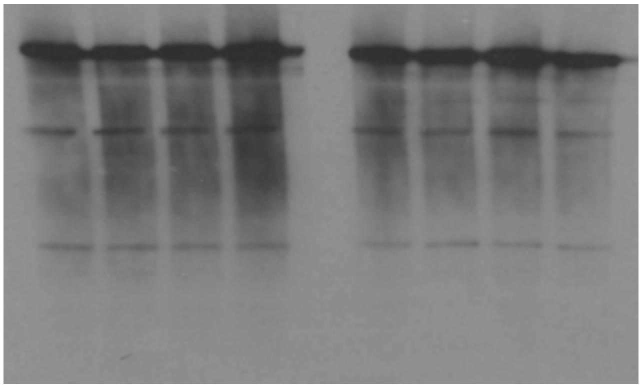

Actin

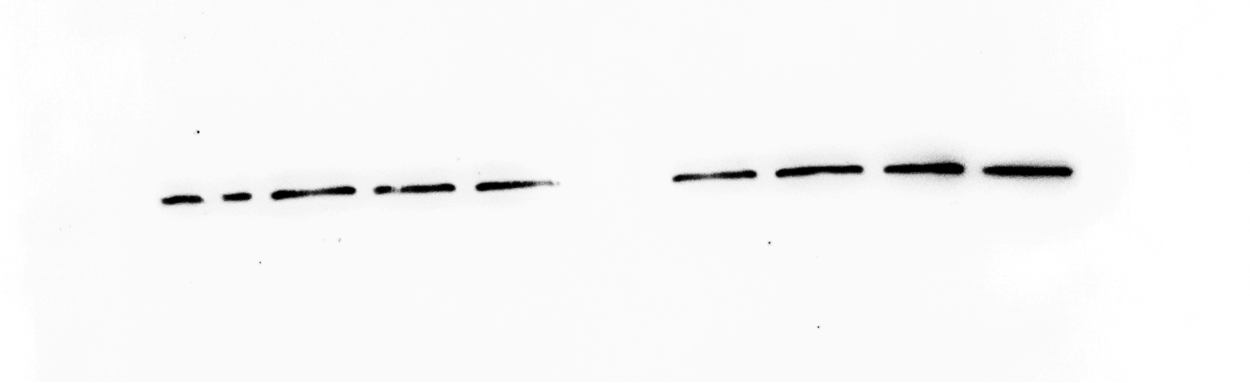

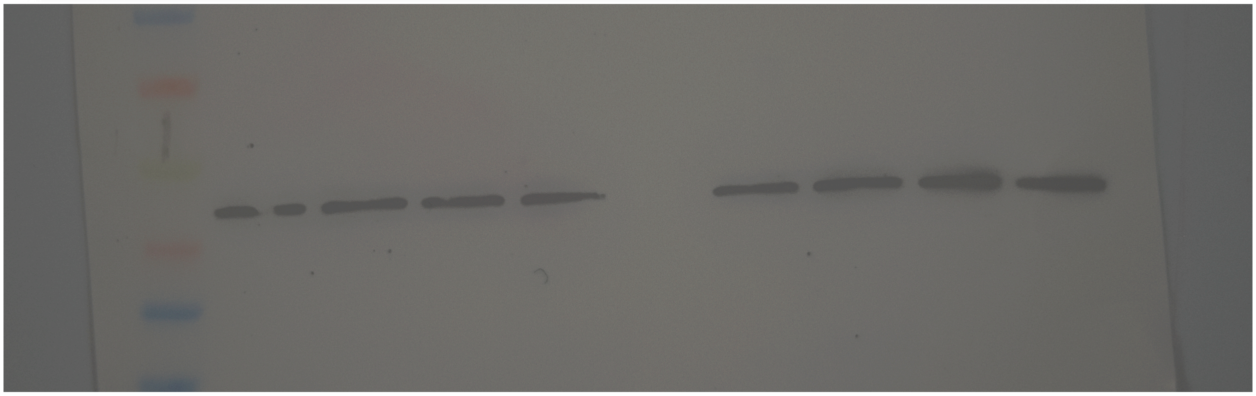

Supplementary Figure 1 B UbK48

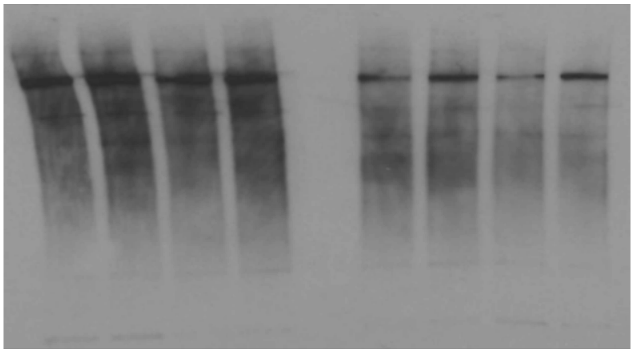

Supplement: Supplementary file 2 — Supplementary Information 2. [file 41598_2021_96540_MOESM2_ESM.pdf]
